# Prediction models for high-grade cervical lesions or worse using machine learning

**DOI:** 10.1016/j.eclinm.2026.103819

**Published:** 2026-03-05

**Authors:** Yunyang Deng, Joakim Dillner, Nicholas Baltzer, Laila Sara Arroyo Mühr, Roxana Merino Martinez, Alexander Ploner, Jiayao Lei, Mark Clements

**Affiliations:** a Department of Medical Epidemiology and Biostatistics, Karolinska Institutet, 171 77, Stockholm, Sweden; b Center for Cervical Cancer Elimination, Department of Clinical Science, Intervention and Technology, Karolinska Institutet, 141 52, Stockholm, Sweden

**Keywords:** Prediction model, High-grade cervical lesions, Cervical cancer, Human papillomavirus, Machine learning, Random forest

## Abstract

**Background:**

This study aimed to improve cervical screening efficiency by developing and validating machine-learning models for predicting high-grade cervical lesions or worse (HCL) risk.

**Methods:**

From Swedish nationwide registers, we included 474,072 women invited to cervical screening in 2016 (split into 80% training and 20% test sets) and 370,105 women invited in 2017 for validation. All women underwent index cytology and/or human papillomavirus (HPV) testing within the recommended interval after age 29. Predictors included screening results (cytology and/or HPV testing), other HPV-related factors, and demographic factors (including age). Four random forest models were trained via 5-fold cross-validation with different predictors: Model 1 (M1) (all predictors), M2 (cytology, HPV testing, age), M3 (HPV testing, other HPV-related factors, and demographic factors), and M4 (HPV testing and age). We computed area under the curves (AUCs) and created plots to depict positive predictive value (PPV) by the number of women intervened.

**Findings:**

In training and test sets, 1-, 3-, and 5-year HCL incidence proportions were 0.25%, 0.68%, and 1.05%, respectively. Cross-validated AUCs were 0.83–0.96 (M1), 0.83–0.96 (M2), 0.91–0.94 (M3), and 0.91–0.93 (M4), depending on the prediction intervals. Similar AUCs were found in the test set. Additionally, the AUCs in the validation set were 0.85–0.95 (M1), 0.85–0.95 (M2), 0.91–0.94 (M3), and 0.92–0.93 (M4). Across all intervals, M1 consistently demonstrated the highest PPV, followed by M2, M3, and M4. For each model, PPVs were lowest for 1-year predictions but comparable at 3 and 5 years.

**Interpretation:**

The models demonstrated strong predictive performance. Evaluating PPVs over the number of invited women provides the potential for risk-stratified screening and clinical utility.

**Funding:**

Vetenskapsrådet, FORTE, Karolinska Institutet, Horizon 2020, and Cancerfonden.

**Research in context**

**Evidence before this study**

We conducted a comprehensive PubMed search from database inception to June 11, 2025, without language restrictions. The search terms included: (“cervical intraepithelial neoplasia” OR “CIN2” OR “CIN3” OR “high-grade squamous intraepithelial lesion” OR “HSIL” OR “high-grade cervical lesion” OR “HCL” OR “cervical cancer” OR “screening”) AND (“prediction model” OR “risk model” OR “machine learning” OR “artificial intelligence” OR “deep learning” OR “random forest” OR “support vector machine”). Studies were eligible if they developed or validated models predicting high-grade cervical lesions or worse (HCL) (cervical intraepithelial neoplasia grade 2 or worse), irrespective of population or setting. Study quality varied; frequent limitations included small sample sizes, lack of external validation, reliance on conventional regression methods, or insufficient consideration of model implementation. No formal meta-analysis was conducted for HCL incidence, but reported area under the curve (AUC) ranged from 0.53 to 0.95.

**Added value of this study**

Our study developed and validated random forest models for predicting HCL in a large, population-based cohort using Swedish national register data. A diverse range of predictors-including cervical screening results, medical histories, and demographic characteristics-was used to construct multiple models addressing different clinical questions, such as the sufficiency of screening alone for HCL prediction and the comparative performance of human papillomavirus testing alone versus its combination with cytology. These findings offer practical guidance for integrating models into cervical cancer screening programs depending on data availability. Furthermore, the study proposes a practical and interpretable use of positive predictive values as a metric for evaluating prediction models in the context of screening augmentation. Focusing on positive predictive values over the number of women invited to screen, it provides a direct link between model output and clinical utility, which is often a gap in applied prediction research.

**Implications of all the available evidence**

Our models provide a theoretical framework for evaluating and comparing prediction models for targeted screening, linking model performance directly to clinical and resource outcomes. This approach supports more efficient, risk-based screening strategies by identifying which models offer the greatest clinical yield within a given capacity, which helps guide the implementation of risk-stratified screening based on the current screening programs.

## Introduction

Globally, cervical cancer was ranked fourth in both cancer incidence and mortality among women in 2022.^1^ Cervical screening, which is effective in the prevention and early detection of cervical precancers/cancers, is one of the three pillars (with vaccination and treatment) in the elimination agenda.^2^ Current cervical screening programs primarily rely on age-based guidelines.^3^ However, with advances in machine learning methods^4^ and comprehensive information on individual screening being available,^3^ risk-stratified screening shows considerable potential. This method would enable a transition from one-size-fits-all strategies to risk-stratified screening, optimizing the screening strategies and reducing unnecessary procedures.^3^^,^^4^ Previously developed prediction models for cervical diseases have shown some potential^3^, ^4^, ^5^, ^6^; however, they generally rely on traditional regression techniques, are based on insufficient sample sizes, or overall lack validation. Additionally, few studies have discussed how the prediction models could be integrated within extant screening programs.

This study aimed to develop and validate machine-learning models to predict the 1-, 3-, and 5-year incidence of high-grade cervical lesions or worse (HCL) based on Swedish nationwide registers. Furthermore, we sought to evaluate model performance through positive predictive values (PPVs), providing a clinically interpretable metric to facilitate the implementation of and decision-making for screening programs.

## Methods

### Data source and study population

We used the nationwide Swedish population and healthcare registers, linked at the individual level.^7^ Cervical cytology, human papillomavirus (HPV) testing, and histology were retrieved from the Swedish National Cervical Screening Registry (NKCx).^8^ HPV vaccination status was sourced from the Swedish HPV Vaccination Register,^9^ National Vaccination Register,^10^ and Prescribed Drug Register.^11^ HPV-related non-inflammatory diseases, HPV-related precancers, and human immunodeficiency virus (HIV) infection were extracted from the National Patient Register.^12^ Genital wart information was obtained from the National Patient Register^12^ and Prescribed Drug Register.^11^ Cancer was identified from the Swedish Cancer Register.^13^ Deaths were collected from the Cause of Death Register.^14^ Parity and smoking during pregnancy were retrieved from the Medical Birth Registry.^15^ Immigration, emigration, birth year, birth country, and county of residence were obtained from the Total Population Register.^16^ Education and income were collected from the Longitudinal Integration Database for Health Insurance and Labor Market Studies.^17^ Finally, women were linked to their parents via the Multi-Generation Register.^18^

**Fig. 1:**
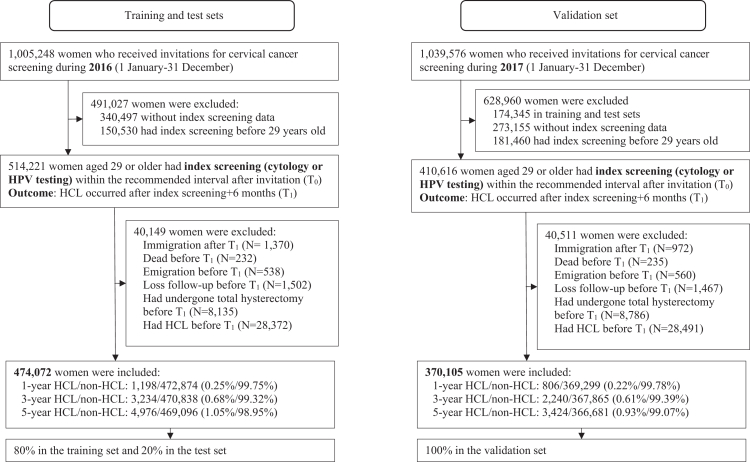
Flow chart of the study population.

For the training and test sets, we first identified 1,005,248 women invited for cervical screening in 2016 (Fig. 1). Of them, 514,221 women underwent an index screening (defined as the first screening following invitation, either cytology or HPV testing) within the recommended interval-3 years for women <50 years, 5 years for those ≥50 years^3^-after the age of 29 (T_0_). The age restriction was used to ensure that all women had the opportunity for at least one screening. The outcome was defined as HCL occurring at least six months after the index screening (T_1_), to ensure the temporal sequence between predictors and HCL and also considering that the index screening tests were likely part of the diagnostic process for HCL rather than preventive screening (Figure S1). We further excluded 40,149 women due to immigration after T_1_; or death, emigration, loss to follow-up, total hysterectomy, or HCL diagnosis before T_1_. Finally, 474,072 women were included, of whom 80% were randomly assigned to the training set and 20% to the test set. For the validation set, 1,039,576 women invited for screening in 2017 were identified. After excluding those already included in training/test sets and applying the same exclusion criteria, 370,105 women remained in the validation set (Fig. 1).

### Outcomes

Outcomes were the 1-, 3-, and 5-year incidence of histologically confirmed HCL, including cervical intraepithelial neoplasia (CIN) grades 2, CIN3, adenocarcinoma in situ or worse (ACIS+), and invasive cervical cancers, identified at least 6 months after the index screening.

### Predictors

Predictors included the following sets (Table S1): (1) Index screening and screening history: Cytology and/or HPV testing. In NKCx, cytology was dichotomized into normal and abnormal, with the latter being defined as atypical squamous cells of undetermined significance (ASCUS) or worse.^19^ HPV testing was categorized as negative or positive for 14 high-risk HPV types (16, 18, 31, 33, 35, 39, 45, 51, 52, 56, 58, 59, 66, and 68). (2) Other HPV-related factors: HPV vaccination status and dose, non-cervical HPV-related precancers/cancers, non-inflammatory diseases of vulva, perineum, and vagina, genital warts (and episodes), HIV infection, and maternal HCL history. International Classification of Diseases (ICD) codes are listed in Table S2. (3) Demographic factors: Age at index screening, country of birth, county of residence, highest education level, annual household income level, high parity (>4), smoking during pregnancy, maternal birth country, and parental highest education level. Education was categorized into seven levels defined by the Swedish education system.^17^ Income was divided into ten groups based on the income deciles for individuals aged 20–65. We introduced an extra group, “missing”, for any factors with missing values. All factors except age and country of birth were measured the year before the index screening.

To include cytology and HPV testing from the index screening as predictors, women were categorized into three groups (Figure S1): Group A (cytology only): Women who underwent cytology only at index T_0_ without HPV testing within the next 30 days. The 30-day window was applied to exclude HPV tests conducted as part of post-cytology clinical workup or triage. Group B (cytology and HPV testing): Women who received both cytology and HPV testing-either on the same day, or with one test following the other within a 30-day interval. In all instances, the earlier test date was considered the index T_0_. Group C (HPV testing only): Women who underwent HPV testing only at index T_0_, with no cytology within the next 30 days.

Additionally, annual cytological histories before the index screening were included as predictors. HPV testing history was classified as either ever negative or ever positive due to the limited number of tests before 2015.^8^ Furthermore, we calculated several cytology history-related predictors derived from a previous study,^3^ including the number of missed screenings, consecutive number of missed screenings, years of delay beyond the recommended screening schedule, consecutive number of benign screenings, total number of screening events, and the worst cytological result during the screening history (Table S1).

### Statistical analysis

Random forest prediction models for binary outcomes were fitted to the training data via 5-fold cross-validation using the default parameter settings. Four models were developed based on different predictor sets (Figure S1, Table S3): (1) Model 1: cytology, HPV testing, and all other predictors (all 64 predictors). (2) Model 2: cytology, HPV testing, and age (48 predictors). (3) Model 3: HPV testing and all other predictors (19 predictors). (4) Model 4: HPV testing and age (3 predictors). For Models 1 and 2, women from all three previously mentioned groups (Groups A, B and C) were included. For Models 3 and 4, only women with HPV testing results at the index were included (Groups B and C). By comparing Models 1 and 2 (or Models 3 and 4), we can assess whether screening alone, without other HPV-related factors and additional demographic factors, is sufficient to predict HCL. Similarly, comparing Models 1 and 3 (or Models 2 and 4) allows us to evaluate the predictive ability of HPV testing alone versus its combination with cytology.

Area under the curve (AUC) for the four models at 1-, 3-, and 5-year prediction intervals was calculated to evaluate the overall model performance. Additionally, we generated plots to illustrate the PPVs by different number of women invited to screening in the four models within the training and validation sets. We specifically contrasted PPVs at two fixed hypothetical thresholds, a strict one where the top 1000 women with the highest predicted risk would be followed up, and a more liberal one, where the top 10,000 women would be followed up. AUCs and PPVs were reported with standard 95% confidence intervals (CIs) for individual models. Where appropriate, p-values for differences between pairs of models in the training and validation sets were reported based on a non-parametric bootstrap with 3000 resamples.

Importance values for all predictors in each model were assessed using the test data. For each predictor, we calculated the reduction of predictive performance as 1-AUC when omitting the specific predictor from the model, so that higher values indicated greater importance. Normalized predictor importance values were subsequently derived by comparison with 1-AUC for the original models including all predictors as the reference.

We performed a sensitivity analysis to enhance the comparability between Models 1 vs 3 and 2 vs 4, to assess the predictive performance of HPV testing alone versus in combination with cytology. Specifically, AUCs for all models were calculated and compared only among women with both cytology and HPV testing from Group B (Figure S1). As a further sensitivity analysis, we fine-tuned all prediction models via sequential model-based optimization on three parameters (number of predictor variables per tree, proportion of data sampled per tree, size of terminal node).^20^ Performances of fine-tuned models and default models were compared as AUCs. Finally, we calculated AUCs and PPVs with their 95% CIs using conventional logistic regression for the same four models in the test set to establish a baseline for the performance of the machine learning/random forest approaches.

Data management was performed using SAS version 9.4. Data analyses were conducted with R version 4.4.3. Prediction models were fitted and evaluated in the R-based mlr3 framework.^21^ Variable importance was assessed via the DALEX package version 2.4.3.^22^ Details of the random forest setup were described in the Supplementary Methods. All statistical tests were two-sided, with the level of statistical significance set at p < 0.05.

Ethical approval was granted by the Stockholm regional review board (Dnr. 02-556, 2011/921-32, 2023-06456-02), with a waiver for written informed consent. The study followed the “Transparent Reporting of a Multivariable Prediction Model for Individual Prognosis or Diagnosis” (TRIPOD) statement.^23^

### Role of the funding source

The funders had no role in study design, data collection, data analysis, data interpretation, or writing of the report.

## Results

### Characteristics of participants

Among the 474,072 women in the training and test data, the 1-, 3-, and 5-year HCL incidence proportions were 0.25% (1198 cases), 0.68% (3234 cases), and 1.05% (4976 cases), respectively (Fig. 1). Compared to those without 5-year HCL, women with 5-year HCL were younger at index age (36.77 vs 44.27 years), more frequently Nordic-born, had lower rates of high parity, higher rates of smoking during pregnancy, higher proportions of Nordic-born mothers, higher HPV vaccination coverage and doses, lower rates of other HPV-related precancers/cancers, and higher rates of HPV-related non-inflammatory diseases, genital warts, HIV infection, and maternal HCL history. For screening results, women with 5-year HCL exhibited higher rates of abnormal index cytology, positive index HPV testing, and historical positive HPV testing (Table 1). Similar patterns were observed in the population invited for screening in 2017 (the validation set) (Table 1), among women included in Models 3 and 4 (Groups B + C) (Table S4), and among women with and without 1 or 3-year HCL (data not shown).

**Table 1:** Characteristics for all women in the main analysis (Groups A + B + C) by 5-year high-grade cervical lesions.

Variable^a^^,^^b^	Training and test sets			Validation set		
	Overall	Non HCL	HCL	Overall	Non HCL	HCL
Number (%)	474,072	469,096	4976	370,105	366,681	3424
**Demographic factors**						
Age at the index screening, years	44.19 (36.40, 51.77)	44.27 (36.47, 51.82)	36.77 (32.14, 44.02)	44.70 (36.82, 52.55)	44.77 (36.90, 52.61)	36.83 (32.26, 43.70)
Birth country						
Nordic	361,955 (76.35)	357,898 (76.30)	4057 (81.53)	282,458 (76.32)	279,704 (76.28)	2754 (80.43)
Others	112,117 (23.65)	111,198 (23.70)	919 (18.47)	87,647 (23.68)	86,977 (23.72)	670 (19.57)
High parity (>4)						
No	347,749 (73.35)	344,265 (73.39)	3484 (70.02)	270,393 (73.06)	267,999 (73.09)	2394 (69.92)
Yes	8533 (1.80)	8470 (1.81)	63 (1.27)	6081 (1.64)	6051 (1.65)	30 (0.88)
Missing	117,790 (24.85)	116,361 (24.81)	1429 (28.72)	93,631 (25.30)	92,631 (25.26)	1000 (29.21)
Smoking status during pregnancy						
No	275,626 (58.14)	273,005 (58.20)	2621 (52.67)	212,982 (57.55)	211,150 (57.58)	1832 (53.50)
Yes	54,706 (11.54)	53,934 (11.50)	772 (15.51)	38,427 (10.38)	37,963 (10.35)	464 (13.55)
Missing	143,740 (30.32)	142,157 (30.30)	1583 (31.81)	118,696 (32.07)	117,568 (32.06)	1128 (32.94)
Maternal birth country						
Nordic	346,026 (72.99)	342,123 (72.93)	3903 (78.44)	270,313 (73.04)	267,661 (73.00)	2652 (77.45)
Others	28,465 (6.00)	28,121 (5.99)	344 (6.91)	20,928 (5.65)	20,678 (5.64)	250 (7.30)
Missing	99,581 (21.01)	98,852 (21.07)	729 (14.65)	78,864 (21.31)	78,342 (21.37)	522 (15.25)
**Other HPV-related factors**						
HPV vaccination						
No	470,921 (99.34)	465,996 (99.34)	4925 (98.98)	366,806 (99.11)	363,430 (99.11)	3376 (98.60)
Yes	3151 (0.66)	3100 (0.66)	51 (1.02)	3299 (0.89)	3251 (0.89)	48 (1.40)
HPV vaccine dose						
0	470,921 (99.34)	465,996 (99.34)	4925 (98.98)	366,806 (99.11)	363,430 (99.11)	3376 (98.60)
1	575 (0.12)	567 (0.12)	8 (0.16)	579 (0.16)	570 (0.16)	9 (0.26)
2	556 (0.12)	550 (0.12)	6 (0.12)	631 (0.17)	619 (0.17)	12 (0.35)
3	2020 (0.43)	1983 (0.42)	37 (0.74)	2089 (0.56)	2062 (0.56)	27 (0.79)
Other HPV-related precancers and cancers^c^	450 (0.09)	447 (0.10)	3 (0.06)	340 (0.09)	335 (0.09)	5 (0.15)
Non-inflammatory diseases of vulva, perineum, and vagina^c^	13,208 (2.79)	13,040 (2.78)	168 (3.38)	10,941 (2.96)	10,820 (2.95)	121 (3.53)
Genital warts						
No	225,322 (47.53)	221,844 (47.29)	3478 (69.90)	169,024 (45.67)	166,630 (45.44)	2394 (69.92)
Yes	11,117 (2.35)	10,837 (2.31)	280 (5.63)	9206 (2.49)	8986 (2.45)	220 (6.43)
Missing	237,633 (50.13)	236,415 (50.40)	1218 (24.48)	191,875 (51.84)	191,065 (52.11)	810 (23.66)
Episode of genital warts						
0	225,322 (47.53)	221,844 (47.29)	3478 (69.90)	169,024 (45.67)	166,630 (45.44)	2394 (69.92)
1	9259 (1.95)	9020 (1.92)	239 (4.80)	7679 (2.07)	7495 (2.04)	184 (5.37)
2	416 (0.09)	403 (0.09)	13 (0.26)	323 (0.09)	315 (0.09)	8 (0.23)
3	1157 (0.24)	1136 (0.24)	21 (0.42)	946 (0.26)	922 (0.25)	24 (0.70)
≥4	285 (0.06)	278 (0.06)	7 (0.14)	258 (0.07)	254 (0.07)	4 (0.12)
Missing	237,633 (50.13)	236,415 (50.40)	1218 (24.48)	191,875 (51.84)	191,065 (52.11)	810 (23.66)
HIV infection^c^	705 (0.15)	690 (0.15)	15 (0.30)	496 (0.13)	486 (0.13)	10 (0.29)
Maternal HCL history^c^	20,672 (4.36)	20,306 (4.33)	366 (7.36)	15,909 (4.30)	15,670 (4.27)	239 (6.98)
**Index screening and screening history**						
Cytology at the index screening						
Normal	327,754 (69.14)	324,702 (69.22)	3052 (61.33)	199,662 (53.95)	197,660 (53.91)	2002 (58.47)
Abnormal	15,164 (3.20)	13,489 (2.88)	1675 (33.66)	11,578 (3.13)	10,380 (2.83)	1198 (34.99)
Missing	131,154 (27.67)	130,905 (27.91)	249 (5.00)	158,865 (42.92)	158,641 (43.26)	224 (6.54)
History of cytology-related factors						
Number of missed screenings	0.56 ± 1.07	0.56 ± 1.08	0.54 ± 0.98	0.39 ± 0.93	0.39 ± 0.93	0.28 ± 0.77
Consecutive number of missed screenings	1.39 ± 1.20	1.39 ± 1.20	1.20 ± 1.10	1.26 ± 1.13	1.26 ± 1.13	0.99 ± 1.04
Exceeded years since recommended screening interval	2.36 ± 3.98	2.36 ± 3.98	2.22 ± 3.63	1.76 ± 3.67	1.76 ± 3.68	1.18 ± 2.94
Consecutive number of benign results	5.07 ± 2.97	5.09 ± 2.97	3.90 ± 2.56	5.32 ± 3.01	5.33 ± 3.01	4.18 ± 2.63
Total number of screening events	5.35 ± 3.13	5.36 ± 3.13	4.27 ± 2.79	5.63 ± 3.19	5.64 ± 3.19	4.54 ± 2.81
The worst result						
Normal	386,698 (81.57)	382,912 (81.63)	3786 (76.09)	301,529 (81.47)	298,868 (81.51)	2661 (77.72)
Abnormal	51,517 (10.87)	50,697 (10.81)	820 (16.48)	42,634 (11.52)	42,098 (11.48)	536 (15.65)
Missing	35,857 (7.56)	35,487 (7.56)	370 (7.44)	25,942 (7.01)	25,715 (7.01)	227 (6.63)
High-risk HPV at the index screening						
No	150,612 (31.77)	150,325 (32.05)	287 (5.77)	179,802 (48.58)	179,512 (48.96)	290 (8.47)
Yes	14,480 (3.05)	12,958 (2.76)	1522 (30.59)	14,926 (4.03)	13,570 (3.70)	1356 (39.60)
Missing	308,980 (65.18)	305,813 (65.19)	3167 (63.65)	175,377 (47.39)	173,599 (47.34)	1778 (51.93)
High-risk HPV history						
No	156,463 (33.00)	156,103 (33.28)	360 (7.23)	183,091 (49.47)	182,760 (49.84)	331 (9.67)
Yes	16,053 (3.39)	14,469 (3.08)	1584 (31.83)	16,767 (4.53)	15,400 (4.20)	1367 (39.92)
Missing	301,556 (63.61)	298,524 (63.64)	3032 (60.93)	170,247 (46.00)	168,521 (45.96)	1726 (50.41)
Group						
Group A. Cytology only						
Cytology only	275,354 (58.08)	273,274 (58.26)	2080 (41.80)	148,158 (40.03)	147,037 (40.10)	1121 (32.74)
Cytology date < HPV testing date & interval >30 days	33,626 (7.09)	32,539 (6.94)	1087 (21.84)	27,219 (7.35)	26,562 (7.24)	657 (19.19)
Group B. Cytology and HPV testing						
Cytology date = HPV testing date	32,001 (6.75)	30,484 (6.50)	1517 (30.49)	33,787 (9.13)	32,430 (8.84)	1357 (39.63)
Cytology date < HPV testing date & interval ≤ 30 days	1329 (0.28)	1297 (0.28)	32 (0.64)	1540 (0.42)	1485 (0.40)	55 (1.61)
Cytology date > HPV testing date & interval ≤ 30 days	608 (0.13)	597 (0.13)	11 (0.22)	536 (0.14)	526 (0.14)	10 (0.29)
Group C. HPV testing only						
HPV testing only	125,368 (26.44)	125,232 (26.70)	136 (2.73)	151,677 (40.98)	151,533 (41.33)	144 (4.21)
Cytology date > HPV testing date & interval >30 days	5786 (1.22)	5673 (1.21)	113 (2.27)	7188 (1.94)	7108 (1.94)	80 (2.34)

HCL, high-grade cervical lesion; HPV, human papillomavirus; HIV, human immunodeficiency virus.

a The characteristics were reported as median (interquartile range) for age, mean ± standard deviation for other continuous variables, and frequency (percentage) for categorical variables.

b Data on county of residence, education, and income were not presented due to the excessive number of categories.

c For binary variables with two levels (“yes” or “no”), only the “yes” category was displayed.

### Overall model performance

**Fig. 2:**
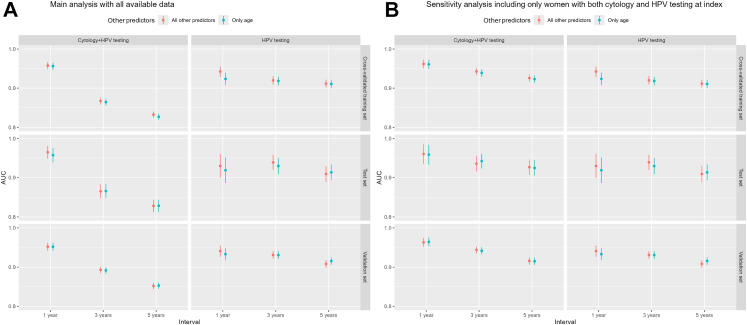
Area under the curve (AUC) for all four models, for the main analysis using all available data for each model (Panel A) and the sensitivity analysis including only women with both cytology and human papillomavirus (HPV) testing at index date (Panel B). In each panel, AUC values and confidence intervals are shown on the vertical axes of the sub-plots, and prediction intervals on the horizontal sub-axes. Models are distinguished by sub-plot columns and colors: sub-plots in the left column show Models 1 + 2, which use both cytology and HPV testing, sub-plots in the right column show Models 3 + 4, which only use HPV testing data. Within each sub-plot, models are distinguished by color, red indicating a model that uses all available register data on top of cervical screening data (Models 1 and 3, respectively) and cyan indicating a model that only uses age at screening as additional information (Models 2 and 4, respectively).

Cross-validated AUCs (95% CIs) in the training data for 1-, 3-, and 5-year intervals in Model 1 were 0.96 (0.95–0.97), 0.87 (0.86–0.88), and 0.83 (0.82–0.84), respectively. In Model 2, the corresponding AUCs (95% CIs) were 0.96 (0.95–0.97), 0.86 (0.86–0.87), and 0.83 (0.82–0.83), respectively. In Model 3, the AUCs (95% CIs) for the 1-, 3-, and 5-year intervals were 0.94 (0.93–0.96), 0.92 (0.91–0.93), and 0.91 (0.90–0.92), respectively. In Model 4, the corresponding AUCs (95% CIs) were 0.92 (0.91–0.94), 0.92 (0.91–0.93), and 0.91 (0.90–0.92), respectively. These findings were consistent in the test data (Fig. 2, Table S5). In the training data, most Models 1 vs 2 and 3 vs 4 comparisons showed no significant differences, except for higher AUC of Model 1 vs 2 at 5 years (p = 0.015) and Model 3 vs 4 at 1 year (p < 0.001). Comparisons of Models 1 vs 3 and 2 vs 4 indicated that Models 1 and 2 performed better at 1 year but underperformed at 3 and 5 years (all p < 0.05) (Table S6).

In the validation data, Model 1 yielded AUCs (95% CIs) of 0.95 (0.94–0.96), 0.89 (0.88–0.90), and 0.85 (0.84–0.86) for the 1-, 3-, and 5-year intervals, respectively. Model 2 showed similar performance, with AUCs of 0.95 (0.94–0.96), 0.89 (0.88–0.90), and 0.85 (0.85–0.86). For Model 3, the corresponding AUCs were 0.94 (0.93–0.95), 0.93 (0.92–0.94), and 0.91 (0.90–0.92), while Model 4 achieved AUCs of 0.93 (0.92–0.95), 0.93 (0.92–0.94), and 0.92 (0.91–0.92) (Fig. 2, Table S5). Most Models 1 vs 2 and Models 3 vs 4 comparisons showed no significant differences, except for a higher AUC for Model 3 vs 4 at 5 years (p = 0.005). Models 1 and 2 generally performed better at 1 year but poorer at 3 and 5 years (all p < 0.05), except for the 1-year comparison between Models 1 and 3 (Table S7).

In the sensitivity analysis, AUCs remained unchanged after fine-tuning (Table S8). When restricted to women with both cytology and HPV testing, the AUCs for the 3- and 5-year intervals in Models 1 and 2 were consistently higher than for the complete data, and either comparable or slightly higher than Models 3 and 4 on the same restricted data. For Model 1, the cross-validated AUCs (95% CIs) for the 1-, 3-, and 5-year intervals were 0.96 (0.95–0.97), 0.94 (0.93–0.95), and 0.93 (0.92–0.93), respectively. In Model 2, the corresponding AUCs (95% CIs) were 0.96 (0.95–0.97), 0.94 (0.93–0.95), and 0.92 (0.91–0.93), respectively (Fig. 2, Table S6, Table S7, Table S9). Furthermore, logistic regression models yielded comparable AUCs to the random forest models in the test set (Table S10).

### Predictive performance for additional screening

**Fig. 3:**
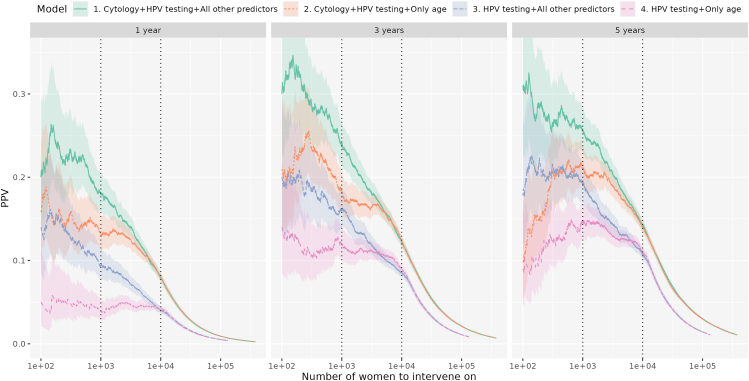
Positive predictive value (PPV) with increasing number of women intervened in the cross-validated training set. PPVs (on the vertical axis) are shown as a function of the number of women intervened (on the horizontal axis), with separate panels for the three prediction intervals. Within each panel, Models 1–4 are distinguished by color. The shaded areas represent 95% confidence intervals. Plausible scenarios discussed in the text (intervention for n = 1000 women and n = 10,000 women) are indicated as dotted vertical lines. We only show PPVs for n ≥ 100, as estimates for smaller numbers were highly unstable.

The cross-validated PPVs ranged from just under 35% for Model 1 at the 3-year prediction with approximately 200 women targeted, to consistently low levels (<0.01) once the number of women intervened approached 100,000. Between these extremes, the cross-validated PPVs of Model 1 and Model 2 gradually converged, as did those of Model 3 and Model 4 (Fig. 3). For instance, with 1000 targeted women, PPVs showed a decreasing trend, ranging from 0.18 (95% CI: 0.16–0.21) for Model 1 to 0.04 (95% CI: 0.03–0.05) for Model 4 at the 1-year outcome, from 0.24 (95% CI: 0.21–0.27) to 0.12 (95% CI: 0.10–0.14) at the 3-year outcome, and from 0.26 (95% CI: 0.23–0.28) to 0.14 (95% CI: 0.12–0.17) at the 5-year outcome, with Models 2 and 3 yielding intermediate values. At the 10,000-women threshold, PPVs were lower across all time points compared with the 1000-women threshold. PPVs were very similar between Models 1 and 2, and likewise between Models 3 and 4. Across models, PPVs ranged from 0.08 (Models 1 and 2) to 0.04 (Models 3 and 4) for the 1-year outcome, from 0.12 to 0.08 for the 3-year outcome, and from 0.14 to 0.11 for the 5-year outcome (Table S11, Fig. 3). p-values for PPV comparisons between models are presented in Tables S6 and S7, with most values < 0.05.

When comparing cross-validated PPVs with those in the validation set, the former generally exhibited higher values, except for Model 4 at the 1-year interval and for Models 2–4 at the 3- and 5-year intervals when fewer than 800 women were intervened (Figure S2). Furthermore, logistic regression models had generally lower, and often dramatically lower PPVs than the random forest models (Table S10).

### Variable importance

**Fig. 4:**
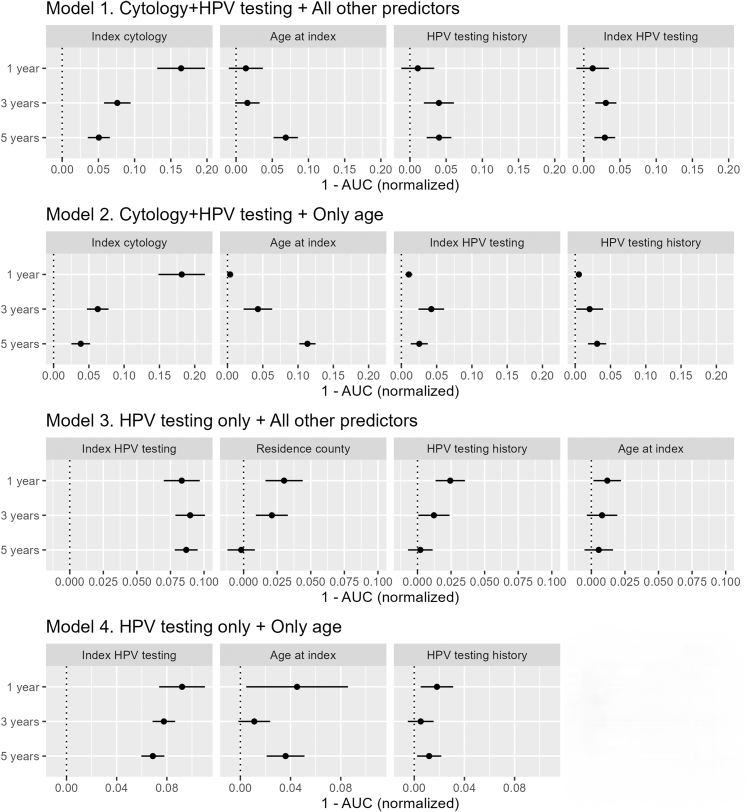
Normalized variable importance values for the top four predictors in the test set. Different panels show the importance of the top four (Models 1–3) or top three (Model 4) predictor variables across all models. In each sub-plot, the normalized 1-area under the curve (AUC) value shows the individual contribution of the predictor relative to 1-AUC for the full model (indicated as dotted line at zero) on the horizontal axis, greater values indicating greater importance. Importance values at the three different prediction intervals are shown on the vertical axis.

The top four predictors with the highest normalized variable importance in the test set for Models 1 and 2 were index cytology, index age, HPV testing history, and index HPV testing. In Model 3, the county of residence was additionally identified as a top-four predictor. The ranking of these predictors varied depending on the length of prediction interval. For Model 4, which only included three predictors, the rank of predictors was index HPV testing, index age, and HPV testing history (Fig. 4). The complete lists of importance values are presented in Figures S3–S6. For Model 1, apart from the top four predictors, genital warts and county of residence were the next most important predictors, followed by cytological history and its related variables. For Model 2, the total number of screening events and cytological history were also important predictors apart from the top four. For Model 3, mother's birth country and income emerged as additional key predictors beyond the top four.

## Discussion

In this study, we demonstrated that random forest models integrating cervical screening results, HPV-related factors, and demographic variables exhibited strong discriminatory power for predicting 1-, 3-, and 5-year HCL risks. HPV testing alone showed comparable AUCs to those of combined cytology and HPV testing, especially when restricted to women with both cytology and HPV testing. We evaluated the PPV performance of four predictive models with a focus on their potential integration into cervical cancer screening programs. The model incorporating all predictors (Model 1) yielded the highest PPVs. For all models, PPVs were lowest for 1-year predictions but similar between the 3- and 5-year intervals. All models demonstrated consistent performance across the training, test, and validation sets. Screening results (cytology or HPV testing) and age were identified as the most influential predictors. These findings align with the WHO cervical cancer elimination initiative^2^ by demonstrating the feasibility of machine-learning-driven, risk-stratified screening to enhance early detection of HCL.

Clinically, our models offered a novel and highly practical link between prediction accuracy and screening resource planning through the use of PPV-by-screening-volume curves. This allows screening programs to directly estimate the proportion of HCL detected at different invitation thresholds, enabling data-driven adjustments to screening volumes and follow-up capacity. In addition, the models leveraged rich longitudinal screening histories and national registers, enabling risk stratification, optimization of colposcopy referrals, and flexible implementation across settings with varying infrastructure. These features provided practical support for transitioning toward risk-based cervical screening that could optimize screening intervals, reduce over-screening, and improve resource allocation which will enhance both efficiency and equity in clinical practice.

Previous studies have developed and/or validated predictive models for HCL incidence. A study of 517,884 Estonian women utilized Cox regression to develop predictive models based on reproductive variables and medical history, with AUCs of 0.72 and 0.68 for CIN3+ and cervical cancer, respectively.^24^ A Swedish case–control study of 125,476 women used logistic regression to predict cervical cancer risk based on screening history (64% accuracy, 71% AUC).^2^ A study of 21,720 Chinese women with positive high-risk HPV used random forest to predict CIN3+ based on HPV genotype, epidemiological factors, and pelvic examination (AUC = 0.85).^4^

Our study showed that, for the 3- and 5-year intervals, the AUCs of the cytology + HPV testing models (Models 1 and 2) among all women were lower than those of the HPV testing alone models (Models 3 and 4), which included only women with HPV testing results at index. However, the lower AUCs did not suggest that combining HPV testing with cytology reduces predictive performance compared to using HPV testing alone. The reduced AUCs were mainly attributable to the different study populations, as some participants in Models 1 and 2 lacked cytology or HPV testing data, leading to less accurate predictions. This was supported by the sensitivity analysis restricted to those who received both cytology and HPV testing, which showed a marked improvement in AUCs for Models 1 and 2 at the 3- and 5-year intervals. It is therefore likely the AUC of HPV testing alone is comparable to its combination with cytology. This is consistent with current guidelines that recommend HPV testing as the primary method for cervical cancer screening.^8^

In this study, we found that non-screening HPV-related factors and demographic variables, apart from age, did not substantially influence AUCs. This is not entirely unexpected, as HPV is the primary etiological agent for cervical lesions.^25^ Other factors may serve primarily as proxies or indirect predictors of HPV infection, which is causally linked to the outcome. Furthermore, both HPV exposure and cancer risk are closely associated with age. Specifically, HPV exposure (e.g., sexual activity with different partners) generally decreases with age, whereas cancer risk resulting from HPV infection increases with age. Additionally, HPV vaccination likely had minimal impact on model performance due to its low prevalence in the study population. Women were aged ≥29 in 2016–2017; thus, the benefit from the vaccination program introduced in 2006 is very limited. However, given its proven effectiveness against HCL, HPV vaccination remains a key factor for future modeling.^26^^,^^27^

In addition to AUCs, we presented PPV curves at varying numbers of women intervened to demonstrate the potential integration of our models into the screening program. The proposed augmented framework operates as follows: 1) A woman attends a routine screening at time T_0_; if the result is positive, proceeds with the standard triage and management pathways. 2) If no positive result is observed by time T_1_, a predictive risk score is computed using our predictive models to estimate her probability of developing HCL. 3) A clinical decision threshold (τ) is defined by the program provider, reflecting available resources and risk tolerance. Women with predictive scores below τ continue with routine screening. 4) Women with predictive scores ≥ τ are classified as high-risk and may be offered targeted follow-up interventions, such as intensified screening or modified screening intervals.

Importantly, our findings suggest that prediction performance is interval dependent. PPVs were lowest for the 1-year prediction interval, likely reflecting the residual protective effect of a negative screen at baseline (T_0_), which limited the available signal for short-term risk stratification. Although PPVs were lowest for 1-year predictions, incorporating a short-term interval may still hold clinical relevance by facilitating the identification of women at imminent risk who could benefit from timely follow-up. By contrast, the 3- and 5-year prediction intervals yielded comparable and higher PPVs, indicating that these models remain stable over mid-to long-term horizons, which is reassuring for potential implementation in extended screening intervals.

While PPV is a key indicator of model utility, it is not the sole factor. The extensive register linkage used in Models 1 and 3 presents significant technical and legal challenges for implementation within a screening program. Therefore, their PPV curves should be viewed as best-case scenarios or upper bounds relative to the more feasible Models 2 and 4, which rely solely on predictors already collected routinely in many programs. Moreover, although Model 4 demonstrates a high AUC, its performance in terms of PPV is suboptimal, and thus it is not a viable option for practical implementation. Consequently, Model 2, which includes cytology, HPV testing, and age as predictors, might be the most realistic candidate for consideration. Finally, we emphasize that the current analysis addresses only the relative performance of prediction models within a theoretical screening framework. Assessing the true value of such a framework would require a comprehensive health economic evaluation, including detailed specification of targeted interventions and associated lifetime expected costs and quality-adjusted life-years.

This study has several strengths. First, it has a large sample size and draws on data from high-quality Swedish nationwide registries with virtually complete coverage. Second, we included a rich set of predictors to develop four different models, each with a unique focus. By comparing these models, we evaluated whether screening alone was sufficient to predict HCL. We also assessed the predictive performance of HPV testing alone compared to its combination with cytology. These comparisons provided insights for program providers to adapt the models into practice based on data availability. Third, the random forest algorithm is a robust and effective predictive method that typically performs well with little to no hyperparameter tuning. Fourth, we evaluated both discrimination and PPV across multiple time intervals in combination with various predictors. A screening provider may select a model based on data availability and PPV to aid decision-making, by estimating the number of women who need to be invited for screening to detect a target number of HCL cases-information that can be aligned with existing financial and personnel resources.

However, this study has certain limitations. First, some risk factors for HCL, such as oral contraceptive use, sexually transmitted infections other than genital warts, and lifestyle factors, were not available in the registers and thus were not included, though we have included predictors like socioeconomic factors as proxies.^28^ Additionally, smoking data were only available for women during pregnancy. Nonetheless, given that other HPV-related factors and demographic factors besides age contributed minimally to the models, the impact of these omissions is likely to be small. Second, we developed the models based on a restricted population of women invited for cervical screening in 2016, which may slightly constrain the generalizability of the findings to populations from more recent periods when HPV testing is used as the primary tool for all screened populations. Nevertheless, this specific year was chosen to ensure adequate follow-up duration for prediction and still allow for sufficient number of participants with available HPV testing data, as such data were limited prior to 2015.^8^ Third, we did not have a real external validation as the current validation set consisted of women invited for cervical screening in 2017, one year after the cohort used for the training and test sets. Consequently, external validation in independent populations is recommended.

In conclusion, our study showed that random forest-based models incorporating cervical screening results, HPV-related variables, and demographic characteristics achieved strong predictive performance for 1-, 3-, and 5-year HCL risks. HPV testing alone exhibited predictive capability comparable to combining cytology and HPV testing. Cervical screening results (cytology or HPV testing) and age were the most influential predictors. The development of these models offers valuable support for practitioners in optimizing screening intervals, reducing over-screening, and enhancing the allocation of medical resources.

## Contributors

YD: data curation, formal analysis, investigation, methodology, validation, visualization, writing—original draft, writing—review & editing; JD, NB, LSAM, RMM: investigation, supervision, validation, writing—review & editing; AP: conceptualization, data preparation, programming, formal analysis, methodology, validation, visualization, writing–review and editing; JL, MC: conceptualization, funding acquisition, project administration, supervision, validation, writing—review & editing. YD and JL accessed and verified the underlying study data. All authors read and approved the submitted version.

## Data sharing statement

The raw datasets are not available for sharing because of privacy policies and regulations in Sweden. Additional synthetic data and codes are available on request from the corresponding author for any interested researchers provided all ethical and legal requirements are met.

## Declaration of interests

Joakim Dillner declares grants or contracts from European Union, Gates Foundation, Swedish Cancer Society, Swedish Research Council, Karolinska Institutet funds, Radiumhemmet research funds, National budget of Sweden, Stockholm County Council, Swedish association of local authorities and regions in the past 36 months. Jiayao Lei declares support for the present manuscript from Swedish Cancer Society, Swedish Research Council, Swedish Research Council for Health, Working Life and Welfare, Karolinska Institutet, European Union's Horizon 2020 research and innovation programme, Region Stockholm. Mark Clements declares support for the present manuscript from Swedish Research Council, Swedish Prostate Cancer Foundation, Region Stockholm, Swedish Cancer Foundation, EU Research Council.
