# Supplementary material for: Prediction models for high-grade cervical lesions or worse using machine learning
Source: eClinicalMedicine. 2026 Mar 5;93:103819. doi: 10.1016/j.eclinm.2026.103819 (PMC12972733; doi:10.1016/j.eclinm.2026.103819)
Supplement: Supplementary Figures and Tables [file mmc1.pdf]

Table of contents

Content	Page
Supplementary Methods	2-3
Table S1. Data source and classification of variables used in this study	4-5
Table S2. International Classification of Diseases (ICD) codes used in this study	6
Table S3. Predictors included in four models	7
Table S4. Characteristics for women included in Models 3 and 4 (Groups B+C) by 5-year high-grade cervical lesions	8
Table S5. Area under the curve (AUC) values and 95% confidence intervals (CIs) for four models at 1-, 3-, and 5-year prediction intervals	9
Table S6. Comparisons of model performance in the training data	10
Table S7. Comparisons of model performance in the validation data	11
Table S8. Comparisons of area under the curve (AUC) between base and tuned models	12
Table S9. Area under the curve (AUC) values and 95% confidence intervals (CIs) for four models at 1-, 3-, and 5-year prediction intervals among women with both cytology and HPV testing	13
Table S10. Area under the curve (AUC) values and positive predictive values (PPVs) with 95% confidence intervals (CIs) for four models at 1-, 3-, and 5-year prediction intervals using logistic regression in the test set	14
Table S11. Positive predictive values (PPVs) with 95% confidence intervals (CIs) for four models at 1-, 3-, and 5-year prediction intervals using random forest in the cross-validated training set	15
Figure S1. Timeline, group, and model definitions	16
Figure S2. Positive predictive value (PPV) with increasing number of women intervened in the cross-validated training set and validation set<br>Figure legend: PPVs (on the vertical axis) are shown as a function of the number of women intervened (on the horizontal axis), with separate panels for the three prediction intervals and the four models. Within each panel, cross-validated training set and validation set are distinguished by color. The shaded areas represent 95% confidence intervals. Plausible scenarios discussed in the text (intervention for n=1,000 women vs. n=10,000 women) are indicated as dotted vertical lines. We only show PPVs for n≥100, as estimates for smaller numbers are highly unstable.	17
Figure S3. Variable importance values for all predictors in the test set for Model 1<br>Figure legend: Importance value of each predictor is assessed based on the reduction of predictive performance as 1-AUC when omitting the specific predictor from the model, so that higher values indicate greater importance.	18
Figure S4. Variable importance values for all predictors in the test set for Model 2<br>Figure legend: Importance value of each predictor is assessed based on the reduction of predictive performance as 1-AUC when omitting the specific predictor from the model, so that higher values indicate greater importance.	19
Figure S5. Variable importance values for all predictors in the test set for Model 3<br>Figure legend: Importance value of each predictor is assessed based on the reduction of predictive performance as 1-AUC when omitting the specific predictor from the model, so that higher values indicate greater importance.	20
Figure S6. Variable importance values for all predictors in the test set for Model 4<br>Figure legend: Importance value of each predictor is assessed based on the reduction of predictive performance as 1-AUC when omitting the specific predictor from the model, so that higher values indicate greater importance.	21

Supplementary Methods

The random forest (RF) algorithm is a widely applied machine-learning method that generates predictions by constructing an ensemble ("forest") of decision trees, and combines their predictions, for enhanced stability, accuracy, and generalization compared to individual decision trees. Individual trees are trained on a subset (generally a bootstrap sample) of the dataset, and at each split of the tree, a random subset of features is evaluated for the optimal split; for classification trees like in our case, splits reducing the impurity of nodes are preferred (generally measured via the Gini index). When predicting a new data point, the forest prediction is the majority vote across all individual trees. Training each tree on a different sample reduces variance relative to a single tree; considering only a random subset of features at each node reduces correlation among trees, which reduces the generalization error of the forest. Combining predictions from many moderately correlated trees reduces the problems with overfitting that deep, unpruned decision trees are otherwise known to have.[1]

RF can handle both classification and regression tasks without strong assumptions about data distribution or linearity, it can accommodate high-dimensional data, various types of predictors, and nonlinear interactions, and is robust to noise and moderate amounts of missing data. RF generally works well out-of-the-box, using default values for hyperparameters without model tuning.[2] RF also provides internal estimates of predictor (feature) importance, though these can suffer from bias and have been ignored for our analysis - instead, we adopted a permutation-based model-agnostic variable importance measure.

For our main analysis, we used the mlr3 machine learning framework in v0.22.1,[3] with a RF classification learner implemented by the ranger package v0.17.0. For the main results, we chose the original (uncorrected) Gini index as split criterion, and left all hyperparameters at their ranger-specific default values. The 2016 data was split randomly into 80% training data and 20% test data, stratified on the outcome (1-, 3, or 5-year high-grade cervical lesions or worse) to ensure balance between affected and unaffected women. The cross-validated prediction results for the training data were based on a stratified five-fold data split. Model training was performed on the tensor compute cluster at the Department of Medical Epidemiology and Biostatistics, Karolinska Institutet, using the Slurm scheduling system to manage execution, and the R package future v1.40.0 for parallelization.[4]

As a sensitivity analysis, we also performed hyperparameter tuning of the RF models: we modified the base models from the main analysis to use actual sub-sampling (instead of bootstrapping), and tripled the number of trees in the forest to 1,500, to reduce bias and correlation between trees. Following the recommendations of Probst et al.,[2] we used sequential model-based optimization (SMBO) for three parameters: the number of predictor variables per tree, the proportion of data sampled per tree, and the size of the terminal nodes. The range of possible hyperparameter settings for the number of predictor variables was defined around the square root of the number of predictors, and consequently varied between models; the proportion of data sampled varied between 25% and 75% of the full data, and terminal node size between 1 and 5 observations. Each model was evaluated 100 times, with hyperparameter values determined via SMBO using 3-fold internal cross-validation; model quality was measured via area under the curve (AUC). Model tuning was performed in the same computational environment as the main analysis, using the R packages mlr3tuning[5] v1.3.0 and mlr3mbo v1.1.5.[6]

References:

1. Boulesteix A-L, Janitza S, Kruppa J, König IR. Overview of random forest methodology and practical guidance with emphasis on computational biology and bioinformatics. WIREs Data Mining Knowl Discov 2012;2:493–507 doi: 10.1002/widm.1072.

2. Probst P, Wright MN, Boulesteix A-L. Hyperparameters and tuning strategies for random forest. WIREs Data Mining Knowl Discov. 2019;9:e1301 doi: 10.1002/widm.1301.

3. Lang M, Binder M, Richter J, Schratz P, Pfisterer F, Coors S, et al. “mlr3: A modern object-oriented machine learning framework in R.” Journal of Open Source Software. 2019. doi:10.21105/joss.01903.

4. Henrik Bengtsson. A Unifying Framework for Parallel and Distributed Processing in R using Futures, The R Journal. 2021;13:2:208–227. doi:10.32614/RJ-2021-048.

5. Becker M, Lang M, Richter J, Bischl B. mlr3tuning: Hyperparameter Optimization for mlr3. 2025. doi:10.5281/zenodo.15852621.

6. Becker M, Schneider L, Richter J, Lang M, Bischl B, Pfisterer F, et al. mlr3mbo: Flexible Bayesian Optimization. 2025. https://mlr3mbo.mlr-org.com. Access on 10 December 2025.

Table S1. Data source and classification of variables used in this study

Variable	Classification	Definition	Data source
**1. Study population selection**			
Invitation information	-	-	NKCx
Immigration, emigration, death, and loss of follow-up	Yes or no	-	TNPR
Full hysterectomy	Yes or no	-	NPR
HCL history	Yes or no	See in "2. Outcome"	NKCx
**2. Outcome**			
HCL after 6 months after the index screening (1, 3, 5 years)	Yes or no	"Yes" was defined as histologically confirmed HCL, including cervical intraepithelial neoplasia (CIN) grades 2, CIN3, adenocarcinoma in situ or worse, and invasive cervical cancers	NKCx
**3. Predictor**			
**1) Index screening and screening history**			
Cytology at the index screening	Abnormal, normal, or missing	"Abnormal" was defined as atypical squamous cells of undetermined significance or worse	NKCx
Cytological history (annually)	Abnormal, normal, or missing		NKCx
Cytology history-related factors			
Number of missed screenings	-	Defined as instances where the interval between the invitation date and the most recent previous screening exceeded twice the recommended period, with additional missed screenings noted for longer intervals	NKCx
Consecutive number of missed screenings	-	Consecutive number of missed screenings up to the invitation date, reflecting the rationale that the risk associated with missed screenings might decrease significantly once a new test is performed	NKCx
Exceeded years since recommended screening interval	-	Years of delay beyond the recommended screening schedule, capturing gaps not classified as missed screenings	NKCx
Consecutive number of benign results	-	Representing the count of successive normal cytological results	NKCx
Total number of screening events	-	-	NKCx
The worst cytological result during the screening history	Abnormal, normal, or missing	"Abnormal" was defined as atypical squamous cells of undetermined significance or worse	NKCx
High-risk HPV at the index screening	Yes, no, or missing	"Yes" was defined as positive for 14 high-risk HPV types (16, 18, 31, 33, 35, 39, 45, 51, 52, 56, 58, 59, 66, and 68)	NKCx
High-risk HPV history	Yes, no, or missing		NKCx
**2) Other HPV-related factors**			
HPV vaccination status	Yes or no	"Yes" was defined as receiving at least one dose of the HPV vaccine	SVEVAC, NVR, PDR
HPV vaccine dose	0, 1, 2, or 3	-	SVEVAC, NVR, PDR
History of other HPV-related precancers (carcinoma in situ of the anus and anal canal, vulva, and vagina) and cancers (base of the tongue, tonsil, anus and anal canal, vulva, and vagina)	Yes or no	See "Table S2" for the used International Statistical Classification of Diseases (ICD) codes	NPR, SCR
History of non-inflammatory diseases of vulva, perineum, and vagina	Yes or no		NPR
History of genital warts	Yes, no, or missing		NPR, PDR
Episode of genital warts	0, 1, 2, 3, ≥4, or missing	-	NPR, PDR
History of HIV infection	Yes or no	See "Table S2" for the used ICD codes	NPR
Maternal HCL history (biological)	Yes or no	See in "2. Outcome"	MGR, SCR
**3) Demographic and socioeconomic factors**			
Age at the index screening	-		TNPR
Country of birth	Nordic, others, or missing	Nordic was defined as Sweden, Denmark, Finland, Norway, and Iceland	TNPR
County of residence	Actual county or missing	Blekinge, Dalarna, Gotland, Gävleborg, Halland, Jämtland, Jänköping, Kalmar, Kronoberg, Norrbotte, Skåne, Stockholm, Södermanl, Uppsala, Värmland, Västerbot, Västernor, Västmanla, Västra Gö, örebro, and östergötl	TNPR
Highest education level	Actual education or missing	Was categorized into seven Swedish levels: pre-secondary education of less than 9 years, pre-secondary education of 9 years, upper secondary education of no more than 2 years, secondary education of 3 years, post-secondary education of less than 3 years, post-secondary education of 3 years or more (excluding postgraduate education), and postgraduate education	LISA

Variable	Classification	Definition	Data source
Annual household income level	Deciles or missing	Was divided into ten groups based on income deciles for individuals aged 20-65 years	LISA
High parity (>4)	Yes, no, or missing	-	MBR
Smoking status during pregnancy	Yes, no, or missing	-	MBR
Maternal birth country (adoptive or biological)	Nordic, others, or missing	See in "Birth country"	MGR, LISA
Parental highest education level (adoptive or biological)	Actual education or missing	See in "Highest education level before the index screening"	MGR, LISA

HCL, high-grade cervical lesion or worse; HPV, human papillomavirus; HIV, human immunodeficiency virus; NKCx, Swedish National Cervical Screening Registry; TPR, Total Population Register; NPR, National Patient Register; SVEVAC, Swedish HPV Vaccination Register; NVR, National Vaccination Register; PDR, Prescribed Drug Register; SCR, Swedish Cancer Register; MGR, Multi-Generation Register; LISA, Longitudinal Integration Database for Health Insurance and Labor Market Studies; MBR, Medical Birth Registry.

Table S2. International Classification of Diseases (ICD) codes used in this study

Disease	ICD-10 (1997-)	ICD-9 (1987-1996)	ICD-8 (1969-1986)	ICD-7 (1958-1968)
Carcinoma in situ: Anus and anal canal	D013	2305/230F	-	-
Carcinoma in situ of vulva	D071	2333/233D	-	-
Carcinoma in situ of vagina	D072	2333/233D	-	-
Malignant tumor of base of tongue	C01	1410/141A	1410	1410
Malignant tumor of tonsil	C09	1460/146A	1460	1450
		1461/146B		
		1462/146C		
Malignant tumor of the anus and anal canal	C21	1542/154C	1542	-
		1543/154D		
		2306/230G		
Malignant neoplasm of vulva	C51	1841/184B	1841	1760
		1842/184C		
		1843/184D		
		1844/184E		
Malignant neoplasm of vagina	C52	1840/184A	1840	1761
Other noninflammatory disorders of vulva and perineum	N90	624	-	-
Other noninflammatory disorders of vagina	N89	623	-	-
Genital warts	A630	0781/078B	791	-
Human immunodeficiency virus infection	B20-B24	42	-	-
	D83/D84	279		

HPV, human papillomavirus.

Table S3. Predictors included in four models

Model	1) Index screening and screening history		2) Other HPV-related factors	3) Demographic factors	
	Cytology: index screening, history, and related factors	High-risk HPV: index screening and history		Age at the index screening	Other factors
1. Cytology+HPV testing+All other predictors	√	√	√	√	√
2. Cytology+HPV testing+age	√	√		√	
3. HPV testing+All other predictors		√	√	√	√
4. HPV testing+age		√		√	

HPV, human papillomavirus.

Table S4. Characteristics for women included in Models 3 and 4 (Groups B+C) by 5-year high-grade cervical lesions

Variable [a,b]	Training and test sets			Validation set		
	Overall	Non HCL	HCL	Overall	Non HCL	HCL
Number (%)	165,092	163,283	1,809	194,728	193,082	1,646
**Demographic factors**						
Age at the index screening, years	45·21 (36·71, 53·39)	45·31 (36·78, 53·45)	37·31 (32·43, 45·74)	45·26 (37·11, 53·14)	45·33 (37·19, 53·22)	37·27 (32·59, 44·67)
Birth country						
Nordic	120,917 (73·24)	119,476 (73·17)	1,441 (79·66)	142,971 (73·42)	141,690 (73·38)	1,281 (77·83)
Others	44,175 (26·76)	43,807 (26·83)	368 (20·34)	51,757 (26·58)	51,392 (26·62)	365 (22·17)
High parity (>4)						
No	116,984 (70·86)	115763 (70·90)	1,221 (67·50)	137,988 (70·86)	136,875 (70·89)	1,113 (67·62)
Yes	3,064 (1·86)	3,040 (1·86)	24 (1·33)	3,151 (1·62)	3,133 (1·62)	18 (1·09)
Missing	45,044 (27·28)	44,480 (27·24)	564 (31·18)	53,589 (27·52)	53,074 (27·49)	515 (31·29)
Smoking status during pregnancy						
No	89,971 (54·50)	89,077 (54·55)	894 (49·42)	104,699 (53·77)	103,893 (53·81)	806 (48·97)
Yes	19,769 (11·97)	19,490 (11·94)	279 (15·42)	19,780 (10·16)	19,522 (10·11)	258 (15·67)
Missing	55,352 (33·53)	54,716 (33·51)	636 (35·16)	70,249 (36·08)	69,667 (36·08)	582 (35·36)
Maternal birth country						
Nordic	113,885 (68·98)	112,514 (68·91)	1,371 (75·79)	135,090 (69·37)	133,873 (69·33)	1,217 (73·94)
Others	12,118 (7·34)	11,977 (7·34)	141 (7·79)	13,159 (6·76)	13,003 (6·73)	156 (9·48)
Missing	39,089 (23·68)	38,792 (23·76)	297 (16·42)	46,479 (23·87)	46,206 (23·93)	273 (16·59)
**Other HPV-related factors**						
HPV vaccination						
No	163,905 (99·28)	162,115 (99·28)	1,790 (98·95)	192,792 (99·01)	191,173 (99·01)	1,619 (98·36)
Yes	1,187 (0·72)	1,168 (0·72)	19 (1·05)	1,936 (0·99)	1,909 (0·99)	27 (1·64)
HPV vaccine dose						
0	163,905 (99·28)	162,115 (99·28)	1,790 (98·95)	192,792 (99·01)	191,173 (99·01)	1,619 (98·36)
1	257 (0·16)	253 (0·15)	4 (0·22)	375 (0·19)	369 (0·19)	6 (0·36)
2	221 (0·13)	219 (0·13)	2 (0·11)	386 (0·20)	379 (0·20)	7 (0·43)
3	709 (0·43)	696 (0·43)	13 (0·72)	1,175 (0·60)	1,161 (0·60)	14 (0·85)
Other HPV-related precancers and cancers [c]	205 (0·12)	204 (0·12)	1 (0·06)	200 (0·10)	196 (0·10)	4 (0·24)
Non-inflammatory diseases of vulva, perineum, and vagina [c]	4,924 (2·98)	4,874 (2·99)	50 (2·76)	6,146 (3·16)	6,084 (3·15)	62 (3·77)
Genital warts						
No	73,689 (44·64)	72,487 (44·39)	1,202 (66·45)	86,077 (44·20)	84,975 (44·01)	1,102 (66·95)
Yes	3,842 (2·33)	3,747 (2·29)	95 (5·25)	4,829 (2·48)	4,720 (2·44)	109 (6·62)
Missing	87,561 (53·04)	87,049 (53·31)	512 (28·30)	103,822 (53·32)	103,387 (53·55)	435 (26·43)
Episode of genital warts						
0	73,689 (44·64)	72,487 (44·39)	1,202 (66·45)	86,077 (44·20)	84,975 (44·01)	1,102 (66·95)
1	3,193 (1·93)	3,115 (1·91)	78 (4·31)	4005 (2·06)	3,920 (2·03)	85 (5·16)
2	147 (0·09)	143 (0·09)	4 (0·22)	183 (0·09)	176 (0·09)	7 (0·43)
3	409 (0·25)	398 (0·24)	11 (0·61)	500 (0·26)	485 (0·25)	15 (0·91)
≥4	93 (0·06)	91 (0·06)	2 (0·11)	141 (0·07)	139 (0·07)	2 (0·12)
Missing	87,561 (53·04)	87,049 (53·31)	512 (28·30)	103,822 (53·32)	103,387 (53·55)	435 (26·43)
HIV infection [c]	303 (0·18)	292 (0·18)	11 (0·61)	273 (0·14)	267 (0·14)	6 (0·36)
Maternal HCL history [c]	6,975 (4·22)	6,845 (4·19)	130 (7·19)	8,167 (4·19)	8,047 (4·17)	120 (7·29)
**Index screening and screening history**						
High-risk HPV at the index screening						
No	150,612 (91·23)	150,325 (92·06)	287 (15·87)	179,802 (92·33)	179,512 (92·97)	290 (17·62)
Yes	14,480 (8·77)	12,958 (7·94)	1,522 (84·13)	14,926 (7·67)	13,570 (7·03)	1,356 (82·38)
High-risk HPV history						
No	149,197 (90·37)	148,909 (91·20)	288 (15·92)	177,635 (91·22)	177,354 (91·85)	281 (17·07)
Yes	14,554 (8·82)	13,070 (8·00)	1,484 (82·03)	15,538 (7·98)	14,241 (7·38)	1,297 (78·80)
Missing	1,341 (0·81)	1,304 (0·80)	37 (2·05)	1,555 (0·80)	1,487 (0·77)	68 (4·13)

HCL, high-grade cervical lesion; HPV, human papillomavirus; HIV, human immunodeficiency virus.

[a]: The characteristics were reported as median (interquartile range) for age and frequency (percentage) for categorical variables.

[b]: Data on county of residence, education, and income were not presented due to the excessive number of categories.

[c]: For binary variables with two levels (“yes” and “no”), only the “yes” category was displayed.

Table S5. Area under the curve (AUC) values and 95% confidence intervals (CIs) for four models at 1-, 3-, and 5-year prediction intervals

Model	Interval	AUC (95% CI)		
		**Cross-validated training set**	**Test set**	**Validation set**
1. Cytology+HPV testing+All other predictors	1 year	0·96 (0·95-0·97)	0·96 (0·95-0·98)	0·95 (0·94-0·96)
	3 years	0·87 (0·86-0·88)	0·87 (0·85-0·88)	0·89 (0·88-0·90)
	5 years	0·83 (0·82-0·84)	0·83 (0·81-0·84)	0·85 (0·84-0·86)
2. Cytology+HPV testing+age	1 year	0·96 (0·95-0·97)	0·96 (0·94-0·98)	0·95 (0·94-0·96)
	3 years	0·86 (0·86-0·87)	0·87 (0·85-0·88)	0·89 (0·88-0·90)
	5 years	0·83 (0·82-0·83)	0·83 (0·81-0·84)	0·85 (0·85-0·86)
3. HPV testing+All other predictors	1 year	0·94 (0·93-0·96)	0·93 (0·90-0·96)	0·94 (0·93-0·95)
	3 years	0·92 (0·91-0·93)	0·94 (0·92-0·96)	0·93 (0·92-0·94)
	5 years	0·91 (0·90-0·92)	0·91 (0·89-0·93)	0·91 (0·90-0·92)
4. HPV testing+age	1 year	0·92 (0·91-0·94)	0·92 (0·89-0·95)	0·93 (0·92-0·95)
	3 years	0·92 (0·91-0·93)	0·93 (0·91-0·95)	0·93 (0·92-0·94)
	5 years	0·91 (0·90-0·92)	0·91 (0·89-0·93)	0·92 (0·91-0·92)

HPV, human papillomavirus.

Table S6. Comparisons of model performance in the training data

Data and comparison [a]		AUC	p-value	PPV at k=1,000	p-value	PPV at k=10,000	p-value
**Full data vs screening only, all data - corresponding to data in Table S5**							
Model 1	*Model 2*						
cytoHPV_full_1yr	cytoHPV_scr_1yr	0·958 vs 0·956	5·6e-01	0·180 vs 0·132	2·7e-05	0·080 vs 0·080	3·9e-01
cytoHPV_full_3yrs	cytoHPV_scr_3yrs	0·867 vs 0·864	3·1e-01	0·238 vs 0·181	1·2e-05	0·122 vs 0·123	4·4e-01
cytoHPV_full_5yrs	cytoHPV_scr_5yrs	0·833 vs 0·827	1·5e-02	0·256 vs 0·211	1·0e-03	0·143 vs 0·141	1·3e-01
Model 3	*Model 4*						
HPVonly_full_1yr	HPVonly_scr_1yr	0·942 vs 0·924	2·1e-06	0·094 vs 0·039	2·7e-08	0·041 vs 0·041	9·2e-01
HPVonly_full_3yrs	HPVonly_scr_3yrs	0·920 vs 0·918	5·8e-01	0·160 vs 0·119	2·7e-03	0·085 vs 0·090	4·7e-06
HPVonly_full_5yrs	HPVonly_scr_5yrs	0·911 vs 0·910	7·8e-01	0·192 vs 0·145	1·7e-03	0·107 vs 0·110	5·3e-02
**Cytology + HPV vs only HPV, all data - corresponding to data in Table S5**							
Model 1	*Model 3*						
cytoHPV_full_1yr	HPVonly_full_1yr	0·958 vs 0·942	8·0e-03	0·180 vs 0·094	8·6e-10	0·080 vs 0·041	1·5e-58
cytoHPV_full_3yrs	HPVonly_full_3yrs	0·867 vs 0·920	1·8e-22	0·238 vs 0·160	9·3e-07	0·122 vs 0·085	1·4e-32
cytoHPV_full_5yrs	HPVonly_full_5yrs	0·833 vs 0·911	1·7e-66	0·256 vs 0·192	1·5e-04	0·143 vs 0·107	3·1e-28
Model 2	*Model 4*						
cytoHPV_scr_1yr	HPVonly_scr_1yr	0·956 vs 0·924	6·3e-07	0·132 vs 0·039	2·7e-14	0·080 vs 0·041	4·2e-62
cytoHPV_scr_3yrs	HPVonly_scr_3yrs	0·864 vs 0·918	1·4e-22	0·181 vs 0·119	7·8e-05	0·123 vs 0·090	1·1e-24
cytoHPV_scr_5yrs	HPVonly_scr_5yrs	0·827 vs 0·910	4·3e-73	0·211 vs 0·145	3·9e-05	0·141 vs 0·110	1·6e-21
**Cytology + HPV vs only HPV, overlapping data only - corresponding to data in Table S9**							
Model 1	*Model 3*						
cytoHPV_full_1yr	HPVonly_full_1yr	0·962 vs 0·942	1·4e-04	0·138 vs 0·094	1·4e-04	0·049 vs 0·041	9·0e-08
cytoHPV_full_3yrs	HPVonly_full_3yrs	0·942 vs 0·920	1·9e-08	0·181 vs 0·160	1·1e-01	0·090 vs 0·085	3·1e-02
cytoHPV_full_5yrs	HPVonly_full_5yrs	0·926 vs 0·911	3·7e-05	0·219 vs 0·192	8·0e-02	0·113 vs 0·107	2·2e-02
Model 2	*Model 4*						
cytoHPV_scr_1yr	HPVonly_scr_1yr	0·961 vs 0·924	5·8e-10	0·104 vs 0·039	1·9e-09	0·049 vs 0·041	3·4e-07
cytoHPV_scr_3yrs	HPVonly_scr_3yrs	0·939 vs 0·918	1·4e-06	0·140 vs 0·119	1·2e-01	0·088 vs 0·090	2·6e-01
cytoHPV_scr_5yrs	HPVonly_scr_5yrs	0·923 vs 0·910	5·8e-04	0·175 vs 0·145	4·0e-02	0·112 vs 0·110	4·7e-01

AUC, area under the curve; PPV, positive predictive value; HPV, human papillomavirus.
[a]: Model comparisons followed the Methods section. P-values were derived from bootstrap testing using 3,000 resamples.

Table S7. Comparisons of model performance in the validation data

Data and comparison [a]		AUC	p-value	PPV at k=1,000	p-value	PPV at k=10,000	p-value
**Full data vs screening only, all data - corresponding to data in Table S5**							
Model 1	*Model 2*						
cytoHPV_full_1yr	cytoHPV_scr_1yr	0·952 vs 0·952	9·0e-01	0·098 vs 0·076	1·6e-02	0·064 vs 0·064	8·9e-01
cytoHPV_full_3yrs	cytoHPV_scr_3yrs	0·893 vs 0·892	6·1e-01	0·191 vs 0·160	1·7e-02	0·109 vs 0·107	3·8e-01
cytoHPV_full_5yrs	cytoHPV_scr_5yrs	0·852 vs 0·853	6·4e-01	0·216 vs 0·191	4·5e-02	0·126 vs 0·122	2·2e-02
Model 3	*Model 4*						
HPVonly_full_1yr	HPVonly_scr_1yr	0·941 vs 0·933	2·3e-02	0·071 vs 0·047	7·8e-03	0·036 vs 0·036	8·1e-01
HPVonly_full_3yrs	HPVonly_scr_3yrs	0·931 vs 0·931	9·1e-01	0·139 vs 0·097	9·1e-04	0·083 vs 0·088	2·2e-03
HPVonly_full_5yrs	HPVonly_scr_5yrs	0·908 vs 0·916	5·3e-03	0·161 vs 0·122	4·8e-03	0·104 vs 0·109	7·7e-03
**Cytology + HPV vs only HPV, all data - corresponding to data in Table S5**							
Model 1	*Model 3*						
cytoHPV_full_1yr	HPVonly_full_1yr	0·952 vs 0·941	6·1e-02	0·098 vs 0·071	9·1e-03	0·064 vs 0·036	1·7e-48
cytoHPV_full_3yrs	HPVonly_full_3yrs	0·893 vs 0·931	7·0e-19	0·191 vs 0·139	1·6e-04	0·109 vs 0·083	1·1e-23
cytoHPV_full_5yrs	HPVonly_full_5yrs	0·852 vs 0·908	2·4e-48	0·216 vs 0·161	1·9e-04	0·126 vs 0·104	1·4e-16
Model 2	*Model 4*						
cytoHPV_scr_1yr	HPVonly_scr_1yr	0·952 vs 0·933	2·5e-03	0·076 vs 0·047	5·2e-03	0·064 vs 0·036	7·7e-49
cytoHPV_scr_3yrs	HPVonly_scr_3yrs	0·892 vs 0·931	1·6e-19	0·160 vs 0·097	9·2e-06	0·107 vs 0·088	1·1e-13
cytoHPV_scr_5yrs	HPVonly_scr_5yrs	0·853 vs 0·916	1·0e-65	0·191 vs 0·122	7·2e-06	0·122 vs 0·109	2·8e-06
**Cytology + HPV vs only HPV, overlapping data only - corresponding to data in Table S9**							
Model 1	*Model 3*						
cytoHPV_full_1yr	HPVonly_full_1yr	0·963 vs 0·941	2·1e-07	0·086 vs 0·071	1·0e-01	0·044 vs 0·036	6·9e-12
cytoHPV_full_3yrs	HPVonly_full_3yrs	0·944 vs 0·931	2·3e-05	0·172 vs 0·139	8·1e-03	0·090 vs 0·083	1·8e-04
cytoHPV_full_5yrs	HPVonly_full_5yrs	0·916 vs 0·908	6·1e-03	0·191 vs 0·161	2·3e-02	0·109 vs 0·104	1·1e-02
Model 2	*Model 4*						
cytoHPV_scr_1yr	HPVonly_scr_1yr	0·964 vs 0·933	7·0e-12	0·064 vs 0·047	7·3e-02	0·044 vs 0·036	4·4e-10
cytoHPV_scr_3yrs	HPVonly_scr_3yrs	0·941 vs 0·931	6·4e-04	0·141 vs 0·097	1·1e-03	0·087 vs 0·088	6·6e-01
cytoHPV_scr_5yrs	HPVonly_scr_5yrs	0·915 vs 0·916	8·3e-01	0·153 vs 0·122	2·9e-02	0·107 vs 0·109	3·1e-01

AUC, area under the curve; PPV, positive predictive value; HPV, human papillomavirus.

[a]: Model comparisons followed the Methods section. P-values were derived from bootstrap testing using 3,000 resamples.

Table S8. Comparisons of area under the curve (AUC) between base and tuned models [a]

Model	Interval	Cross-validated training set		Test set		Validation set	
		Base	Tuned	Base	Tuned	Base	Tuned
1. Cytology+HPV testing+All other predictors	1 year	0·96	0·96	0·96	0·96	0·95	0·95
	3 years	0·87	0·88	0·87	0·88	0·89	0·90
	5 years	0·83	0·84	0·83	0·84	0·85	0·86
2. Cytology+HPV testing+age	1 year	0·96	0·96	0·96	0·96	0·95	0·95
	3 years	0·86	0·87	0·87	0·87	0·89	0·90
	5 years	0·83	0·83	0·83	0·84	0·85	0·86
3. HPV testing+All other predictors	1 year	0·94	0·95	0·93	0·94	0·94	0·95
	3 years	0·92	0·92	0·94	0·94	0·93	0·94
	5 years	0·91	0·91	0·91	0·92	0·91	0·92

HPV, human papillomavirus.

[a]: Model 4 (HPV testing only) was not tuned due to the limited number of predictors.

Table S9. Area under the curve (AUC) values and 95% confidence intervals (CIs) for four models at 1-, 3-, and 5-year prediction intervals among women with both cytology and HPV testing

Model	Interval	AUC (95% CI)		
		**Cross-validated training set**	**Test set**	**Validation set**
1. Cytology+HPV testing+All other predictors	1 year	0·96 (0·95-0·97)	0·96 (0·94-0·99)	0·96 (0·95-0·97)
	3 years	0·94 (0·93-0·95)	0·94 (0·92-0·96)	0·94 (0·93-0·95)
	5 years	0·93 (0·92-0·93)	0·93 (0·91-0·95)	0·92 (0·91-0·93)
2. Cytology+HPV testing+age	1 year	0·96 (0·95-0·97)	0·96 (0·93-0·98)	0·96 (0·95-0·98)
	3 years	0·94 (0·93-0·95)	0·94 (0·92-0·96)	0·94 (0·93-0·95)
	5 years	0·92 (0·91-0·93)	0·92 (0·90-0·94)	0·91 (0·91-0·92)
3. HPV testing+All other predictors	1 year	0·94 (0·93-0·96)	0·93 (0·90-0·96)	0·94 (0·93-0·95)
	3 years	0·92 (0·91-0·93)	0·94 (0·92-0·96)	0·93 (0·92-0·94)
	5 years	0·91 (0·90-0·92)	0·91 (0·89-0·93)	0·91 (0·90-0·92)
4. HPV testing+age	1 year	0·92 (0·91-0·94)	0·92 (0·89-0·95)	0·93 (0·92-0·95)
	3 years	0·92 (0·91-0·93)	0·93 (0·91-0·95)	0·93 (0·92-0·94)
	5 years	0·91 (0·90-0·92)	0·91 (0·89-0·93)	0·92 (0·91-0·92)

HPV, human papillomavirus.

Table S10. Area under the curve (AUC) values and positive predictive values (PPVs) with 95% confidence intervals (CIs) for four models at 1-, 3-, and 5-year prediction intervals using logistic regression in the test set

Model	Interval	Test set		
		AUC (95% CI)	PPV (95% CI) with 1,000 target women	PPV (95% CI) with 10,000 target women
1. Cytology+HPV testing+All other predictors	1 year	0·95 (0·94-0·96)	0·12 (0·10-0·14)	0·02 (0·02-0·02)
	3 years	0·89 (0·88-0·90)	0·16 (0·13-0·18)	0·04 (0·04-0·05)
	5 years	0·86 (0·85-0·86)	0·18 (0·16-0·21)	0·06 (0·05-0·06)
2. Cytology+HPV testing+age	1 year	0·95 (0·94-0·96)	0·12 (0·11-0·15)	0·02 (0·02-0·02)
	3 years	0·88 (0·87-0·89)	0·14 (0·12-0·17)	0·04 (0·04-0·05)
	5 years	0·85 (0·84-0·85)	0·17 (0·15-0·20)	0·06 (0·05-0·06)
3. HPV testing+All other predictors	1 year	0·96 (0·95-0·97)	0·07 (0·05-0·09)	0·01 (0·01-0·01)
	3 years	0·94 (0·93-0·95)	0·11 (0·10-0·14)	0·03 (0·02-0·03)
	5 years	0·93 (0·92-0·93)	0·14 (0·12-0·16)	0·03 (0·03-0·04)
4. HPV testing+age	1 year	0·93 (0·92-0·94)	0·04 (0·03-0·05)	0·01 (0·01-0·01)
	3 years	0·92 (0·91-0·93)	0·09 (0·08-0·11)	0·03 (0·02-0·03)
	5 years	0·91 (0·90-0·92)	0·12 (0·10-0·14)	0·03 (0·03-0·04)

HPV, human papillomavirus.

Table S11. Positive predictive values (PPVs) with 95% confidence intervals (CIs) for four models at 1-, 3-, and 5-year prediction intervals using random forest in the cross-validated training set

Model	Interval	Cross-validated training set	
		**PPV (95% CI) with 1,000 target women**	**PPV (95% CI) with 10,000 target women**
1. Cytology+HPV testing+All other predictors	1 year	0·18 (0·16-0·21)	0·08 (0·07-0·09)
	3 years	0·24 (0·21-0·27)	0·12 (0·12-0·13)
	5 years	0·26 (0·23-0·28)	0·14 (0·14-0·15)
2. Cytology+HPV testing+age	1 year	0·13 (0·11-0·15)	0·08 (0·08-0·09)
	3 years	0·18 (0·16-0·21)	0·12 (0·12-0·13)
	5 years	0·21 (0·19-0·24)	0·14 (0·13-0·15)
3. HPV testing+All other predictors	1 year	0·09 (0·08-0·11)	0·04 (0·04-0·04)
	3 years	0·16 (0·14-0·18)	0·08 (0·08-0·09)
	5 years	0·19 (0·17-0·22)	0·11 (0·10-0·11)
4. HPV testing+age	1 year	0·04 (0·03-0·05)	0·04 (0·04-0·04)
	3 years	0·12 (0·10-0·14)	0·09 (0·09-0·10)
	5 years	0·14 (0·12-0·17)	0·11 (0·10-0·12)

HPV, human papillomavirus.

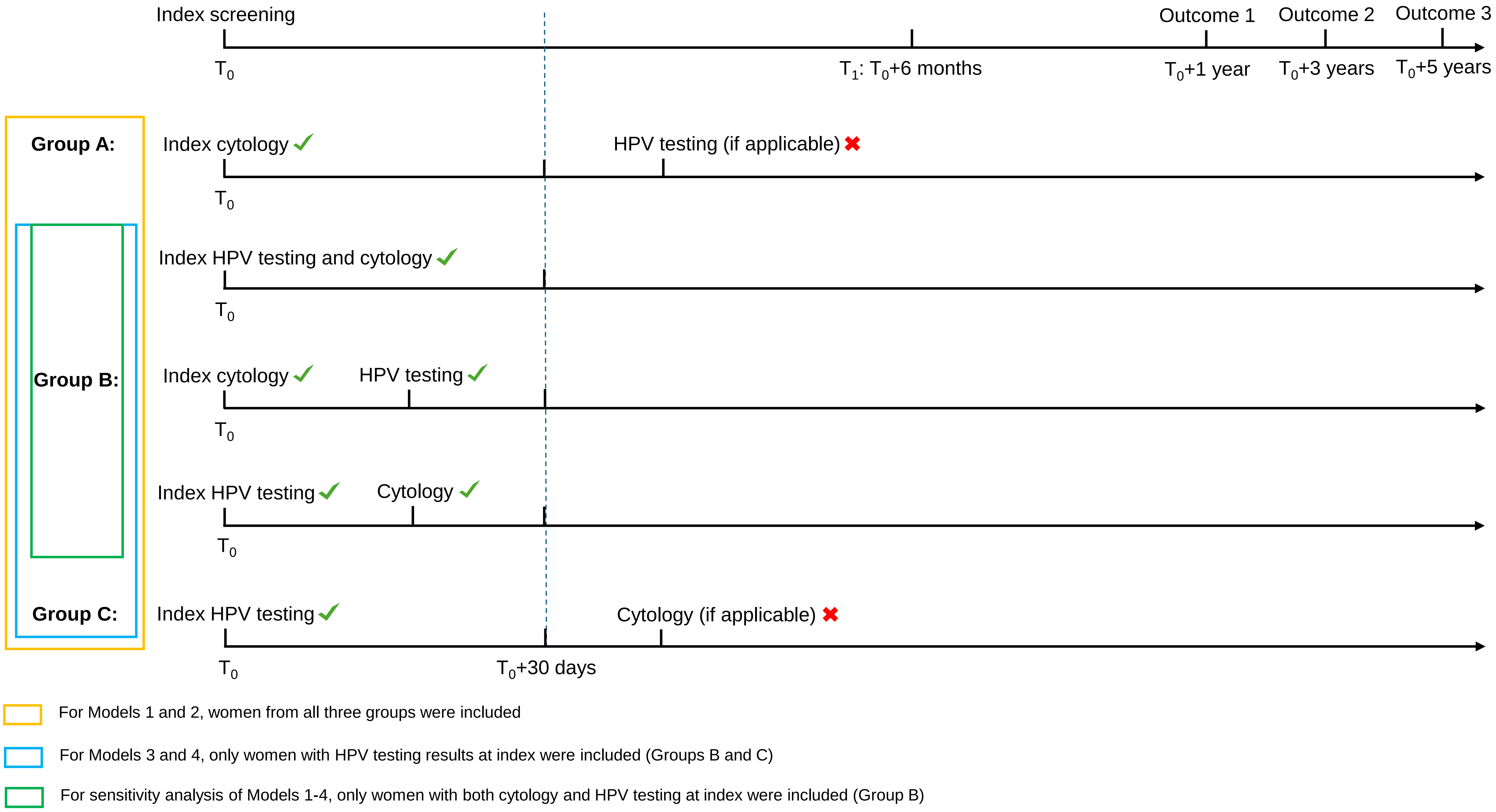

Index screening
Outcome 1
Outcome 2
Outcome 3
T0
T1: T0+6 months
T0+1 year
T0+3 years
T0+5 years
Group A:
Index cytology
HPV testing (if applicable)
T0
Index HPV testing and cytology
T0
Group B:
Index cytology
HPV testing
T0
Index HPV testing
Cytology
T0
Group C:
Index HPV testing
Cytology (if applicable)
T0
T0+30 days
For Models 1 and 2, women from all three groups were included
For Models 3 and 4, only women with HPV testing results at index were included (Groups B and C)
For sensitivity analysis of Models 1-4, only women with both cytology and HPV testing at index were included (Group B)

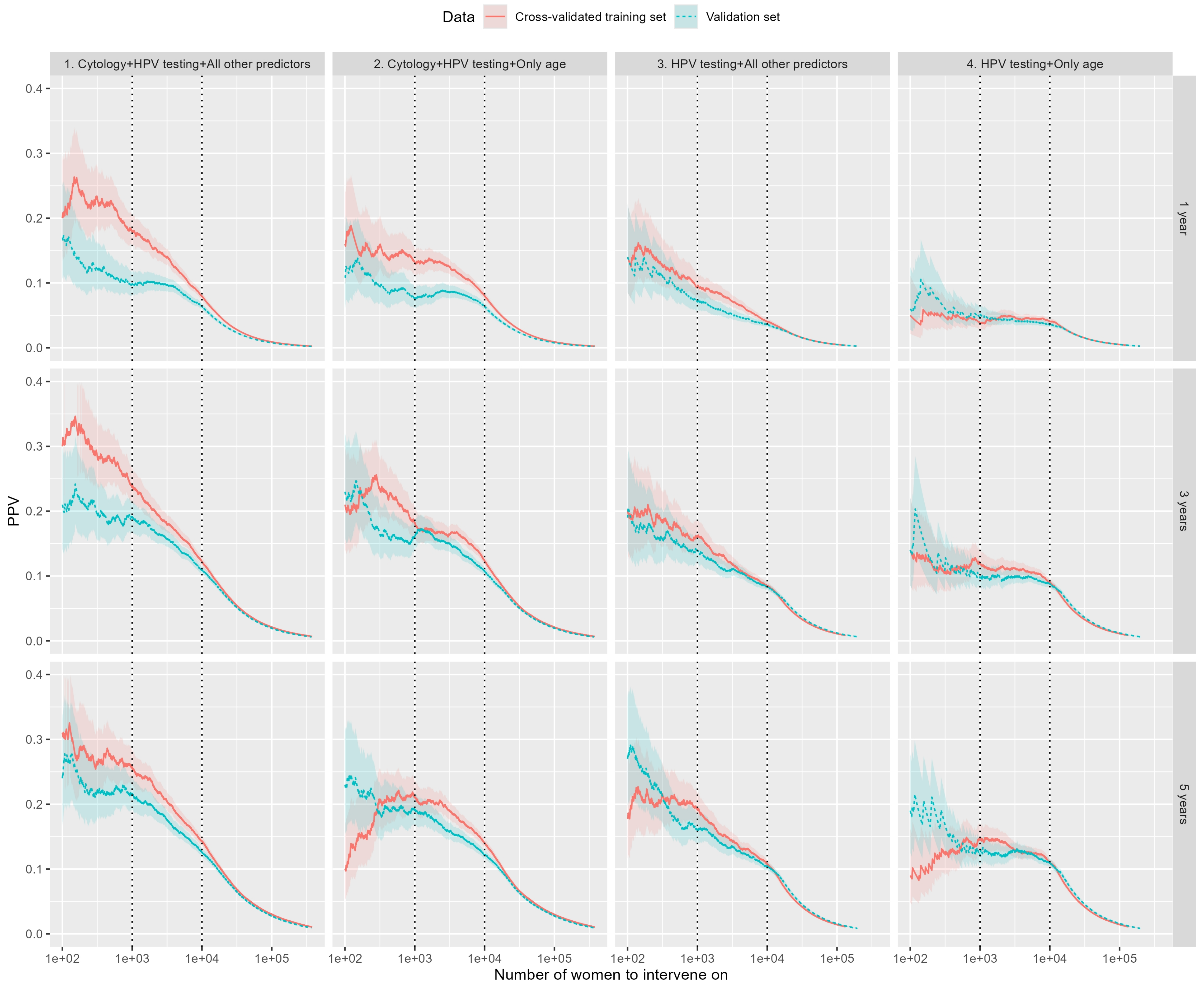

Data
Cross-validated training set
Validation set
1. Cytology+HPV testing+All other predictors
2. Cytology+HPV testing+Only age
3. HPV testing+All other predictors
4. HPV testing+Only age
1 year
3 years
5 years
PPV
0.0
0.1
0.2
0.3
0.4
1e+02
1e+03
1e+04
1e+05
Number of women to intervene on

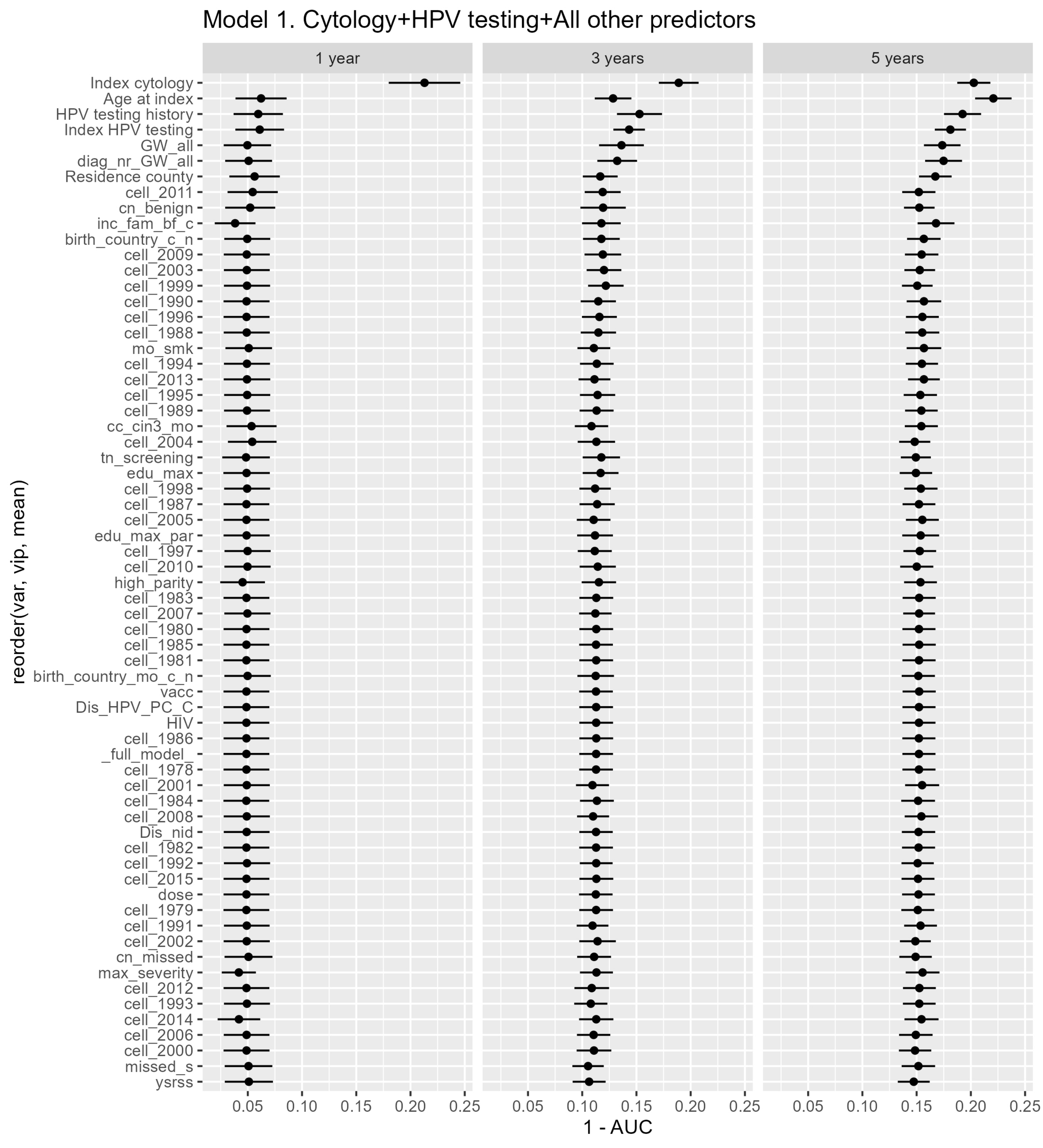

Model 1. Cytology+HPV testing+All other predictors
1 year
3 years
5 years
Index cytology
Age at index
HPV testing history
Index HPV testing
GW_all
diag_nr_GW_all
Residence county
cell_2011
cn_benign
inc_fam_bf_c
birth_country_c_n
cell_2009
cell_2003
cell_1999
cell_1990
cell_1996
cell_1988
mo_smk
cell_1994
cell_2013
cell_1995
cell_1989
cc_cin3_mo
cell_2004
tn_screening
edu_max
cell_1998
cell_1987
cell_2005
edu_max_par
cell_1997
cell_2010
high_parity
cell_1983
cell_2007
cell_1980
cell_1985
cell_1981
birth_country_mo_c_n
vacc
Dis_HPV_PC_C
HIV
cell_1986
_full_model_
cell_1978
cell_2001
cell_1984
cell_2008
Dis_nid
cell_1982
cell_1992
cell_2015
dose
cell_1979
cell_1991
cell_2002
cn_missed
max_severity
cell_2012
cell_1993
cell_2014
cell_2006
cell_2000
missed_s
ysrss
reorder(var, vip, mean)
0.05
0.10
0.15
0.20
0.25
1 - AUC

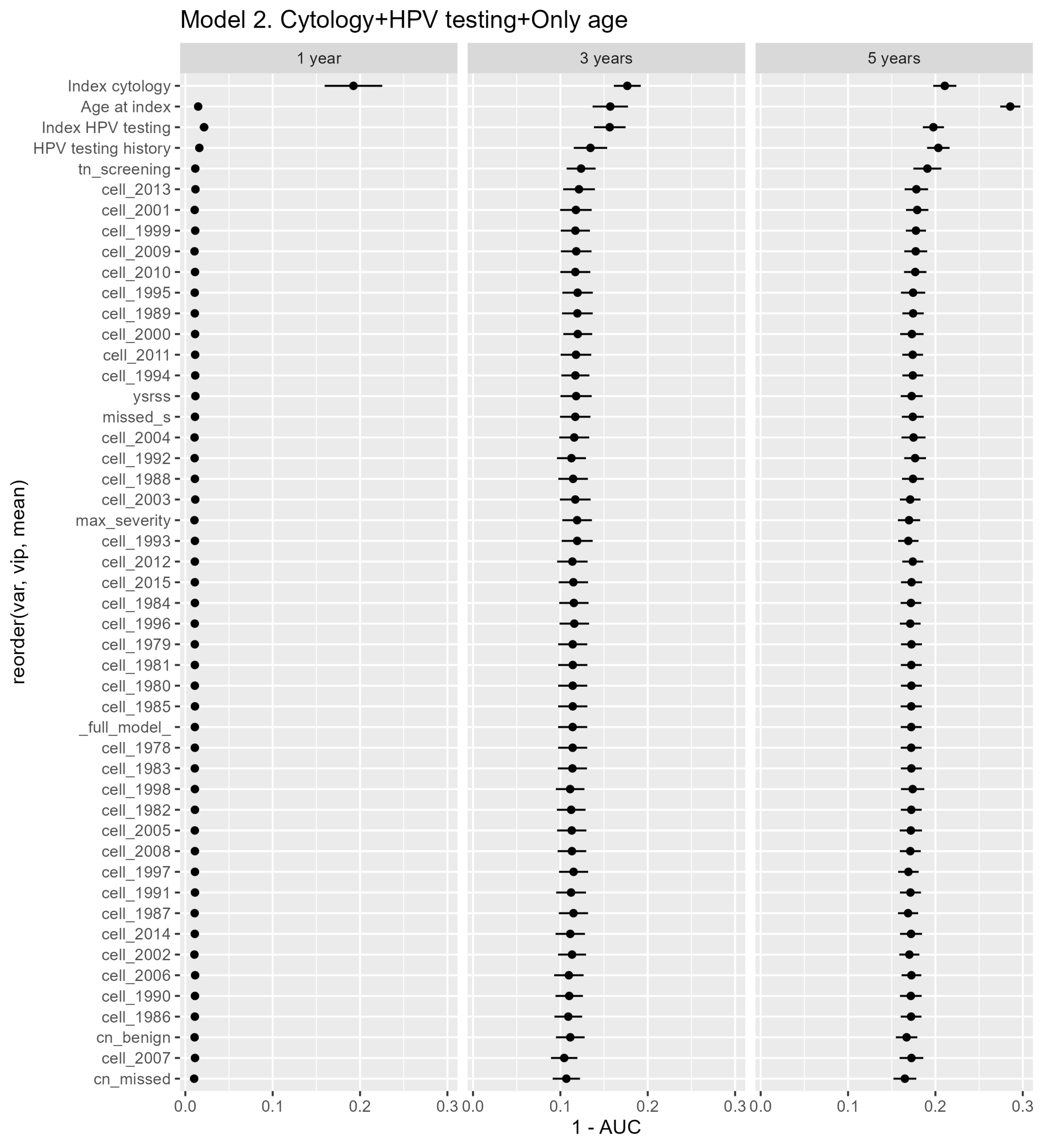

Model 2. Cytology+HPV testing+Only age
1 year
3 years
5 years
Index cytology
Age at index
Index HPV testing
HPV testing history
tn_screening
cell_2013
cell_2001
cell_1999
cell_2009
cell_2010
cell_1995
cell_1989
cell_2000
cell_2011
cell_1994
ysrss
missed_s
cell_2004
cell_1992
cell_1988
cell_2003
max_severity
cell_1993
cell_2012
cell_2015
cell_1984
cell_1996
cell_1979
cell_1981
cell_1980
cell_1985
_full_model_
cell_1978
cell_1983
cell_1998
cell_1982
cell_2005
cell_2008
cell_1997
cell_1991
cell_1987
cell_2014
cell_2002
cell_2006
cell_1990
cell_1986
cn_benign
cell_2007
cn_missed
reorder(var, vip, mean)
0.0 0.1 0.2 0.3
1 - AUC

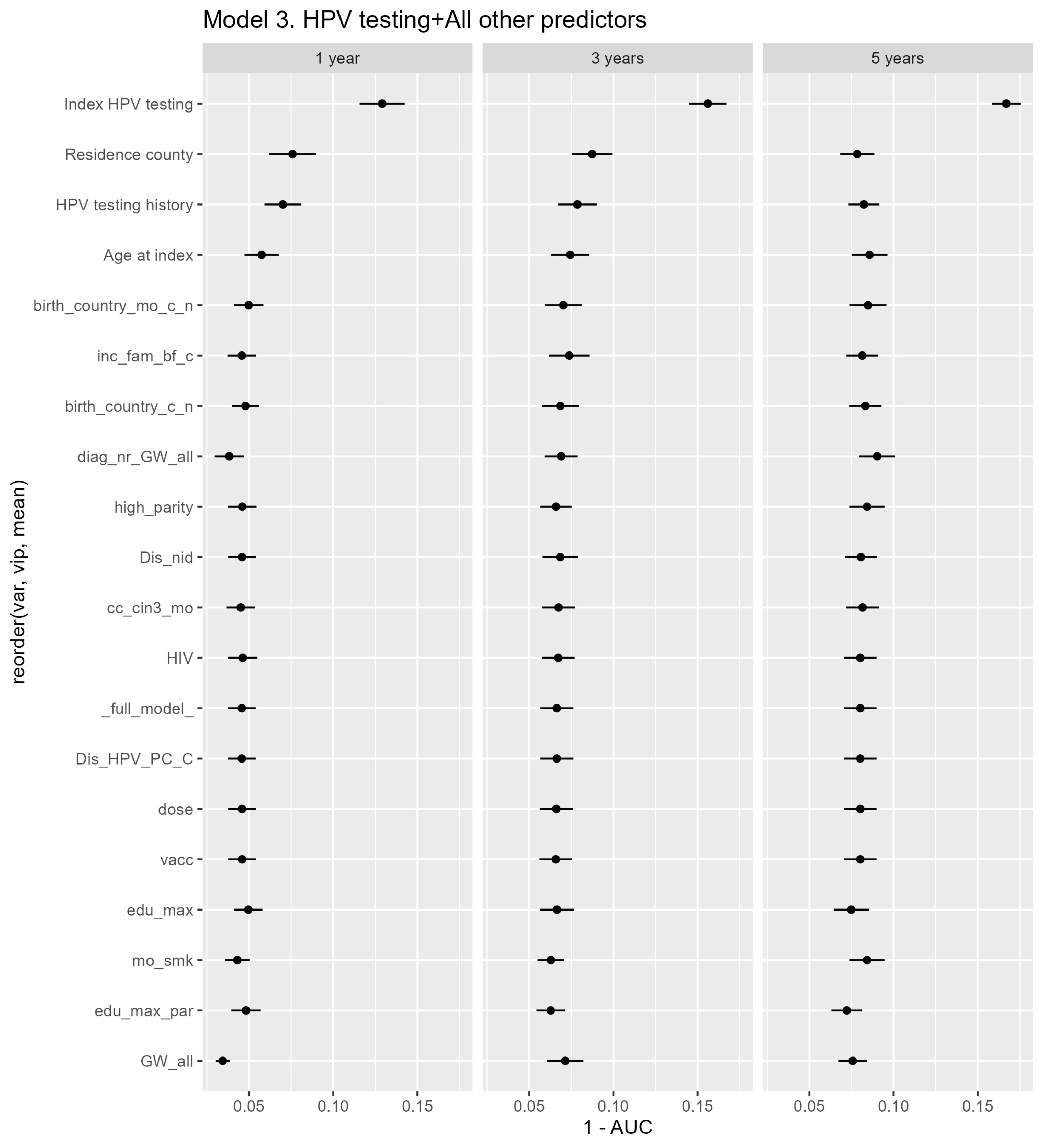
Model 3. HPV testing+All other predictors
1 year
3 years
5 years
Index HPV testing
Residence county
HPV testing history
Age at index
birth_country_mo_c_n
inc_fam_bf_c
birth_country_c_n
diag_nr_GW_all
high_parity
Dis_nid
cc_cin3_mo
HIV
_full_model_
Dis_HPV_PC_C
dose
vacc
edu_max
mo_smk
edu_max_par
GW_all
reorder(var, vip, mean)
0.05
0.10
0.15
1 - AUC

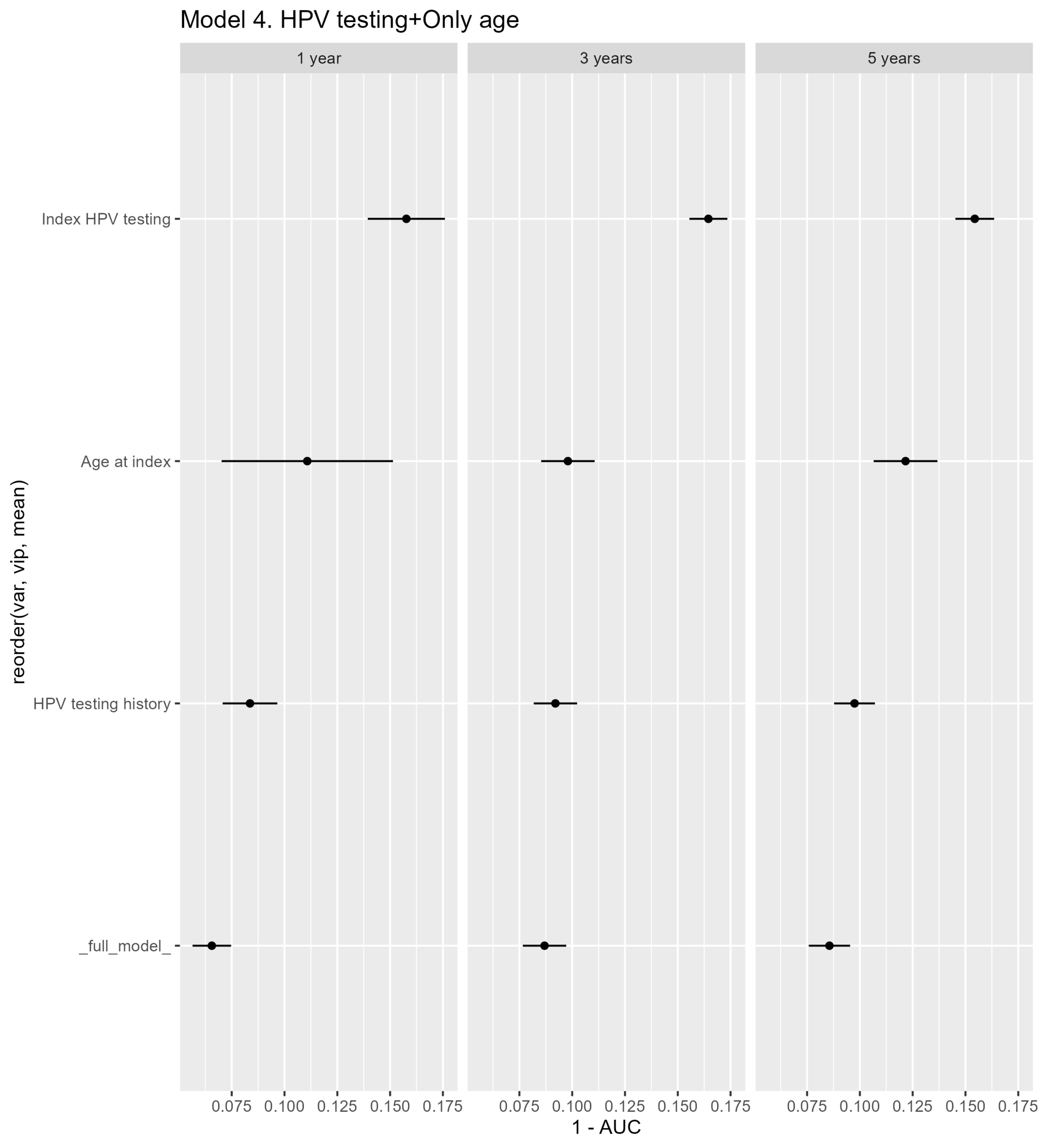
Model 4. HPV testing+Only age
1 year
3 years
5 years
Index HPV testing
Age at index
HPV testing history
_full_model_
reorder(var, vip, mean)
0.075
0.100
0.125
0.150
0.175
1 - AUC
